# The DnaJ Gene Family in Pepper (*Capsicum annuum* L.): Comprehensive Identification, Characterization and Expression Profiles

**DOI:** 10.3389/fpls.2017.00689

**Published:** 2017-05-01

**Authors:** FangFei Fan, Xian Yang, Yuan Cheng, Yunyan Kang, Xirong Chai

**Affiliations:** ^1^College of Horticulture, South China Agricultural UniversityGuangzhou, China; ^2^State key Laboratory Breeding Base for Zhejiang Sustainable Pest and Disease Control, Institute of Vegetables, Zhejiang Academy of Agricultural SciencesHangzhou, China

**Keywords:** DnaJ, heat shock protein 40, chromosomal localization, stress-related *cis*-elements, expression patterns

## Abstract

The DnaJ proteins which function as molecular chaperone played critical roles in plant growth and development and response to heat stress (HS) and also called heat shock protein 40 based on molecular weight. However, little was reported on this gene family in pepper. Recently, the release of the whole pepper genome provided an opportunity for identifying putative DnaJ homologous. In this study, a total of 76 putative pepper *DnaJ* genes (CaDnaJ01 to CaDnaJ76) were identified using bioinformatics methods and classified into five groups by the presence of the complete three domains (J-domain, zinc finger domain, and C-terminal domain). Chromosome mapping suggested that segmental duplication and tandem duplication were occurred in evolution. The multiple stress-related *cis*-elements were found in the promoter region of these *CaDnaJ* genes, which indicated that the CaDnaJs might be involved in the process of responding to complex stress conditions. In addition, expression profiles based on RNA-seq showed that the 47 CaDnaJs were expressed in at least one tissue tested. The result implied that they could be involved in the process of pepper growth and development. qRT-PCR analysis found that 80.60% (54/67) CaDnaJs were induced by HS, indicated that they could participated in pepper response to high temperature treatments. In conclusion, all these results would provide a comprehensive basis for further analyzing the function of CaDnaJ members and be also significant for elucidating the evolutionary relationship in pepper.

## Introduction

With the increase of global warming, high temperature has become one of the most vital abiotic stresses on crop plants (Glazebrook, 1999). Pepper (*Capsicum annuum* L.) which originated in the tropical regions of Latin America had been widely cultivated around the world as an important vegetable crop nowadays and is sensitive to high temperature during plant growth and development, especially in reproductive stage (Guo et al., 2014). The optimum temperature of growing pepper is 20–30°C. Over 32°C can bring about serious effects on pollination and fertilization, and results in blossom and fruit dropping which can cause a significant reduction of pepper fruit yield and quality.

In the long-term evolution, plants have evolved a complicated response mechanism to respond to heat stress (HS). Previous researches had reported that heat shock response (HSR) was induced in many plant species under HS condition (Vierling, 1991). Among them, a great deal of ubiquitous and evolutionary-conserved proteins was identified as heat shock proteins (Hsps), one of the main products of the HSR (Vierling, 1991). The Hsp was first discovered in *Drosophila melanogaster* in response to HS (Ritossa, 1962). In the following years, more Hsps were identified in other plants (Agarwal et al., 2002; Sarkar et al., 2009; Lopes-Caitar et al., 2013; Mulaudzi-Masuku et al., 2015). According to approximate molecular weight and sequence homology, the Hsps were classified into five families, including the Hsp100, Hsp90, Hsp70, Hsp60 and small Hsps (Wang et al., 2004; Kotak et al., 2007; Gupta et al., 2010).

The Hsp40, one of the important plant Hsps, was first identified in *Escherichia coli*, generally existed in organisms as 41 kDa Hsps (Georgopoulos et al., 1980; Bukau and Horwich, 1998; Craig et al., 2006). The Hsp40s, also known as DnaJ proteins or J-proteins, generally consisted of the J-domain, a proximal G/F-domain, a distal zinc finger (CxxCxGxG) domain, and followed by less conserved C-terminal sequences (Caplan et al., 1993; Silver and Way, 1993). The characteristic feature of the J-proteins was the presence of evolutionarily conserved J-domain which located nearby the N-terminus and composed of approximately 70 amino acids residues (Cyr et al., 1992). The invariant tripeptide (HPD) was the hallmark of J-domain. It stimulated the ATPase activity of Hsp70 and was crucial for keeping J-protein’s function (Kampinga and Craig, 2010). Previously, Cheetham and Caplan (1998) attempted to separate these proteins into three groups. Group I J-proteins were characterized by the J-domain, G/F-domain, and zinc finger domain. Group II would have the J-domain plus either a G/F-domain or zinc finger domain. Group III J-proteins only comprised the J-domain (Ohtsuka and Hata, 2000).

In recent years, it has been found that plant DnaJ proteins played important roles in response to both biotic and abiotic stresses, such as pest, pathogenic bacterium, drought, salt, and heat. In 2007, a J-domain virulence effecter of *Pseudomonas syringae* remolded host chloroplasts when responded to pathogen (Jelenska et al., 2007). The researchers reported that over-expression of tomato (*Solanum lycopersicum*) chloroplast-targeted J-protein, *LeCDJ1*, facilitated heat tolerance in transgenic tomatoes (Kong et al., 2014a) and further found that it also played important role in maintaining photosystem II under chilling stress (Kong et al., 2014b). Subsequently, the study has also demonstrated that this gene could enhance tolerance to drought stress and resistance to *P. solanacearum* in transgenic tobacco (Wang et al., 2014). In addition, Xia et al. (2014) reported that a putative J-proteins ortholog from *Nicotiana tabacum* could be involved in drought stress response and its over-expression enhanced drought tolerance possibly through regulating expression of stress-responsive genes.

Up to now, many J-proteins in organisms were identified, such as *Arabidopsis thaliana* (89) (Miernyk, 2001), yeast (22) (Walsh et al., 2004) and human (41) (Craig et al., 2006). Despite ongoing efforts to characterize the members of J-protein from other organisms (Sarkar et al., 2009; Kong et al., 2014a,b; Xia et al., 2014), none of its from pepper has been identified at the genomic level. Fortunately, the pepper whole genomic sequences were completely available (Kim et al., 2014; Qin et al., 2014), which provided an opportunity for identifying candidate J-protein genes at the genomic level. In the present work, the J-protein gene family members were identified in pepper through bioinformatics method and analyzed by integration of gene structure, conserved motifs, chromosomal localization, *cis*-element and expression patterns.

## Materials and Methods

### Genome-Wide Identification of *CaDnaJ* Genes in Pepper

The genomic sequences of pepper downloaded from the Pepper Genome Database (PGD^1^) (Kim et al., 2014) were used to build the local database on the software BioEdit 7.0. The Hidden Markov Model (HMM) profile of J-domain (PF00226) downloaded from the Pfam protein family database^2^ was used as query sequence to search against putative pepper J-protein genes with *e*-value <10^-5^. Subsequently, each of all putative pepper J-protein genes was used to identify the presence of J-domain on Pfam^3^. The protein sequences of identified pepper J-protein gene family members were analyzed with EXPASY PROTOPARAM^4^ to obtain molecular weight and theoretical isoelectric point (pI).

### Multiple Alignment and Chromosomal Location

In this paper, the J-proteins in pepper were classified based on structural features. In each class, the full amino acid sequences of pepper J-proteins were aligned using the software Clustal X 2.01 (Larkin et al., 2007). Each of the J-protein genes was mapped on chromosomes using MapDraw2.1 (Liu and Meng, 2003) based on information in PGD. Two duplication events, tandem duplication and segmental duplication, were also further elaborated. For tandem duplication, three criteria were adopted. Firstly, two or more pepper *DnaJ* genes were arrayed within a range of 100 kb distance. Secondly, the multiple alignments of these *DnaJ* genes had a high coverage rate of the longer gene (more than 70%). Thirdly, the identity of the aligned region in these *DnaJ* genes was also more than 70% (Li et al., 2009; Huang et al., 2012; Wei et al., 2016). The segmental duplication was investigated according to Plant Genome Duplication Database (PGDD^5^).

### Promoters Analysis of Pepper *CaDnaJ* Genes

The upstream regions (1.5 kb) of the *DnaJ* gene sequences were downloaded from PGD, and were used to search for regulating factor such as gibberellins (GA), abscisic acid (ABA), salicylic acid (SA), ethylene, drought, salt and heat.

### Tissue-Specific Expression of *CaDnaJ* Genes Based on RNA-Seq

In this paper, RNA-seq data reported by previous researchers (Kim et al., 2014) were used to investigate expression patterns of putative *CaDnaJ* genes in pepper. Different tissues were selected: root, stem, leaf and pericarp at 6 days post-anthesis (DPA), 16 DPA, 25 DPA, respectively, and mature green (MG), breaker (B), 5 days post-breaker (B5), B10. RPKM (Reads Per Kilo bases per Million mapped Reads) values of *CaDnaJ* genes were log2-transformed (Wei et al., 2012). Heat maps of *CaDnaJ* genes in different tissues were performed using software MultiExperiment Viewer (MeV) (Howe et al., 2010).

### Plant Materials and Heat Stress Treatment

A hot pepper hybrid (*zhejiao 3#*), which developed by Zhejiang Academy of Agricultural Sciences, was selected in the experiments. Seeds were sterilized for 5 min using 10% hypochlorous acid solution and washed three times using distilled water. These seeds were further placed in water-saturated filter paper to germinate, then cultivated in Hoagland solution in a growth chamber which was maintained at a 16 h light at 26°C and 8 h dark at 19°C. At the stage of 6–8 true leaves, plants were treated with 42°C for 4 h and plants grown at 25°C were used as the control group. Young leaves were collected and immediately frozen with liquid nitrogen for total RNA extraction. Each treatment was conducted with three biological replicates, and samples from five plants were collected for each replicate.

### RNA Extraction and qRT-PCR Analysis

Total RNA was extracted using Total RNA kit (Tiangen Biotech, Beijing, China) and reverse-transcribed using FastQuant RT Kit (Tiangen Biotech, Beijing, China), the operational procedure followed the manufacturer’s procedure.

For quantitative RT-PCR analysis, we amplified PCR products in triplicate using 2 × Taq Master Mix (Vazyme, Nanjing, China) in 20 μL qRT-PCR reactions. PCR was performed using the ABI step-one plus 96-well real-time PCR Detection System (Bio-Rad) and cycling conditions consisted of denaturation at 94°C for 5 min, followed by 33 cycles of denaturation at 94°C for 30 s, annealing at 55°C for 30 s and extension at 72°C for 30 s. The *UBI* gene was used as an internal control (Wan et al., 2011). Gene-specific primers were designed and used for amplification as described in **Supplementary Table S1**. Analysis of relative gene expression data was performed using the 2^-ΔΔct^ method (Livak and Schmittgen, 2001).

## Results

### Genome-Wide Identification of *CaDnaJ* Genes in Pepper

A total of 85 putative sequences of pepper *DnaJ* genes were gotten from PGD by HMM search. Among them, nine sequences without a complete J-domain (CA03g32700, CA06g22620, CA00g87730, CA12g09540, CA02g21540, CA05g10320, CA11g03100, CA06g00080, and CA05g07590) were removed. The remaining 76 genes were assigned as pepper *DnaJ* genes. As a matter of convenience, the 76 DnaJ proteins were named as CaDnaJ01 to CaDnaJ76 according to their location on chromosome (**Table 1**).

**Table 1:** The list of CaDnaJ members identified in pepper.

Gene name	Locus name	Location	Chr.	Group^a^	Size(aa)	MW(Da)	PI	Introns
CaDnaJ01	CA01g16030	97618202–97621808	1	C	574	64187.51	8.54	5
CaDnaJ02	CA01g17770	143624907–143627243	1	C	778	32485.78	6.35	0
CaDnaJ03	CA01g18690	156550800–156555986	1	A	300	32485.78	6.35	11
CaDnaJ04	CA01g22020	169879103–169880683	1	C	526	58220.93	8.34	0
CaDnaJ05	CA01g25030	203653187–203656231	1	C	1014	113869.47	5.73	0
CaDnaJ06	CA01g27370	222188570–222189529	1	C	288	32533.02	8.65	1
CaDnaJ07	CA01g30060	252896608–252899637	1	C	968	110547.4	8.45	2
CaDnaJ08	CA02g03340	46401318–46404099	2	C	469	51784.11	7.97	3
CaDnaJ09	CA02g06030	81542166–81542741	2	C	184	20655.63	8.95	1
CaDnaJ10	CA02g07560	109106142–109111419	2	C	505	57920.91	8.19	4
CaDnaJ11	CA02g15460	144816870–144819496	2	B	352	38501.82	9.17	2
CaDnaJ12	CA03g00750	1443148–1444782	3	C	180	21530.33	9.49	1
CaDnaJ13	CA03g08730	28341344–28342355	3	C	249	28757.26	5.52	1
CaDnaJ14	CA03g19380	212945408–212947600	3	C	730	81652.23	9.02	0
CaDnaJ15	CA03g24080	228862136–228863317	3	C	393	43430.55	9.85	0
CaDnaJ16	CA03g25800	235233410–235241868	3	C	785	59082.25	7.21	9
CaDnaJ17	CA03g31950	249441998–249454817	3	A	421	45593.07	9.23	7
CaDnaJ18	CA03g37040	257676539–257678405	3	C	215	23745.93	5.02	5
CaDnaJ19	CA04g03850	11909825–11910715	4	A	286	31473.8	8.86	1
CaDnaJ20	CA04g12150	170393420–170395215	4	C	209	23712.34	5.97	2
CaDnaJ21	CA04g16150	205132799–205133939	4	C	347	37710.14	8.68	2
CaDnaJ22	CA04g16480	205935059–205935586	4	C	175	20694.67	9.97	0
CaDnaJ23	CA04g19270	214481447–214481839	4	E	130	15006.93	9.52	0
CaDnaJ24	CA04g21880	219689981–219690469	4	C	162	17672.7	9.87	0
CaDnaJ25	CA05g00380	478405–487668	5	C	1432	157852.71	8.6	10
CaDnaJ26	CA05g03820	10149653–10153690	5	A	417	46743.88	6.12	5
CaDnaJ27	CA05g09770	104367131–104370250	5	C	212	23288.3	5.32	5
CaDnaJ28	CA05g10040	111541112–111547755	5	E	258	28711.63	8.68	1
CaDnaJ29	CA05g11830	157117370–157119724	5	C	784	88043.88	7.08	0
CaDnaJ30	CA05g12050	165924883–165925699	5	C	183	20601.3	9.21	2
CaDnaJ31	CA05g17350	225414158–225414685	5	C	175	20366.33	9.6	0
CaDnaJ32	CA05g18040	227747673–227748459	5	C	233	27032.68	6.54	1
CaDnaJ33	CA05g19550	231614733–231618044	5	A	419	46582.74	6.01	4
CaDnaJ34	CA06g00330	381222–387261	6	B	345	39132.52	6.35	9
CaDnaJ35	CA06g19300	218954099–218961832	6	B	345	38203.22	9.11	1
CaDnaJ36	CA06g27020	234692516–234693273	6	D	216	24361.61	9.77	1
CaDnaJ37	CA07g03000	13658626–13660185	7	E	519	57652.71	5.32	0
CaDnaJ38	CA07g04780	39783049–39785325	7	C	758	84482.15	8.38	0
CaDnaJ39	CA07g14410	212076067–212082495	7	B	345	37215.12	9.17	2
CaDnaJ40	CA07g14580	212629401–212635278	7	C	295	33432.67	5.6	8
CaDnaJ41	CA07g20780	230038893–230039930	7	C	138	15577.42	9.69	1
CaDnaJ42	CA07g20790	230043861–230044460	7	C	199	16176.37	8.87	0
CaDnaJ43	CA07g21520	231469760–231475634	7	C	562	64225.13	8.53	8
CaDnaJ44	CA08g04550	83124279–83126597	8	C	772	86114.31	8.13	0
CaDnaJ45	CA08g04600	84686555–84694315	8	C	1272	140416.01	6.06	7
CaDnaJ46	CA08g06460	119037486–119039876	8	B	323	35771.63	8.74	2
CaDnaJ47	CA08g09710	127054669–127056621	8	C	650	74087.37	8.35	0
CaDnaJ48	CA08g11000	129804829–129805596	8	C	255	28904.03	9.37	0
CaDnaJ49	CA08g11250	130268529–130271774	8	C	1081	120431.12	8.59	0
CaDnaJ50	CA08g12000	131886063–131888726	8	C	268	29611.44	6.97	6
CaDnaJ51	CA08g12400	132522159–132523390	8	C	169	19029.27	5.96	4
CaDnaJ52	CA08g15850	138087951–138093573	8	A	447	48202.89	9.29	6
CaDnaJ53	CA08g16300	138809830–138812058	8	E	742	81179.23	8.8	0
CaDnaJ54	CA08g16570	139457347–139458186	8	C	279	31836.41	7.73	0
CaDnaJ55	CA09g01340	2706279–2712890	9	C	666	74144.61	5.07	10
CaDnaJ56	CA09g02890	7558262–7563055	9	C	437	48590.89	6.07	7
CaDnaJ57	CA09g10060	137889893–137892146	9	C	524	57233.33	9.24	4
CaDnaJ58	CA09g11550	183732592–183739583	9	B	344	37544.53	9.23	2
CaDnaJ59	CA10g18490	226999515–226999919	10	C	134	15596.59	9.45	0
CaDnaJ60	CA10g20680	230815988–230820272	10	C	285	32803.76	5.92	7
CaDnaJ61	CA10g21560	232003695–232006477	10	C	303	34356.11	7.71	2
CaDnaJ62	CA11g00780	1313193–1320773	11	C	414	45782.29	5.93	10
CaDnaJ63	CA11g05830	31452436–31455481	11	B	309	34460.23	9.18	1
CaDnaJ64	CA11g10010	113882240–113883699	11	B	355	39775.51	7.59	2
CaDnaJ65	CA11g15800	246441094–246443635	11	A	420	46685.7	5.96	5
CaDnaJ66	CA11g15990	246907971–246916099	11	C	249	29550.83	9.42	8
CaDnaJ67	CA12g07660	35330989–35333794	12	A	420	46676.65	6.17	5
CaDnaJ68	CA12g15900	210077200–210078225	12	C	341	38582.29	8.38	0
CaDnaJ69	CA12g16980	217402887–217403441	12	C	184	20599.06	4.81	0
CaDnaJ70	CA12g18310	225521850–225529944	12	C	366	41110.67	5.41	8
CaDnaJ71	CA12g21480	233476472–233477521	12	C	349	40840.83	7.63	0
CaDnaJ72	CA00g32600			A	421	47162.33	7	6
CaDnaJ73	CA00g54170			E	711	77402.78	5.83	0
CaDnaJ74	CA00g57050			C	193	22938.37	4.56	4
CaDnaJ75	CA00g75210			C	771	86305.23	9.37	0
CaDnaJ76	CA00g93240			C	570	62812.03	4.95	7

*^a^Group A are characterized by the J-domain, zinc finger domain and a less conserved C-terminal; Group B are characterized by the J-domain and a less conserved C-terminal; Group C only comprised the J-domain; Group D would both have the J-domain and zinc finger domain; Group E was described as J-like proteins contained a J-protein lacked of HPD motif. MW, Molecular weight; pI, isoelectric points.*

The length of CaDnaJ proteins ranged from 130 (CaDnaJ23) to 1272 (CaDnaJ45) amino acids, and the predicted molecular weights were between 15.597 kDa (CaDnaJ59) and 157.85 kDa (CaDnaJ25). The CaDnaJs shared a conserved J-domain comprised about 70 amino acids, in which CaDnaJ57 owned the shortest J-domain with 39 amino acids, while J-domain of CaDnaJ25 was the longest (84 amino acids). The predicted pI-values of CaDnaJ proteins ranged from 4.56 (CaDnaJ74) to 9.87 (CaDnaJ24), indicating acidic and alkaline proteins. Besides, it was also found that 25 (32.89%) of the total 76 *CaDnaJ* genes had no introns, 11 genes (14.47%) had a single intron, while only CaDnaJ03 had 11 introns (**Table 1**).

### Classification and Sequence Alignment of *CaDnaJ* Genes

The **Ca**DnaJ protein usually contains conserved J-domain, zinc finger domain, and uncharacterized C-terminal domain (Cheetham and Caplan, 1998). According to the presence of the complete three domains, the *CaDnaJ* genes were classified into five groups (A, B, C, D, and E), including 9, 8, 53, 1 and 5 members, respectively. Group A CaDnaJ proteins are characterized by the J-domain, zinc finger domain and a less conserved C-terminal. The difference between Group A and B was lack of the zinc finger domain. Group C CaDnaJ proteins only comprised the J-domain, otherwise, Group D would both have the J-domain and zinc finger domain. Group E contains a CaDnaJ protein lacked of HPD motif, which have been described as J-like proteins (Walsh et al., 2004).

Based on the classification above, sequence alignment of *CaDnaJ* genes was performed separately (**Supplementary Table S2**). It was found that nine members in group A possessed a conserved HPD motif in J-domain and two zinc finger domains (CxxCxGxG). Eight members in group B were lack of the zinc finger domain but owned conserved HPD motif in J-domain. The largest group (group C) which included 53 members only consisted of complete J-domain. Group D which contained merely one member (CaDnaJ36) had both the conserved HPD motif and zinc finger domain. The last group (group E), which comprised five members, held the least conservation among five groups. All the members in group E possessed J-domains lacked of HPD motif.

### Chromosomal Location and Gene Duplication

**FIGURE 1:**
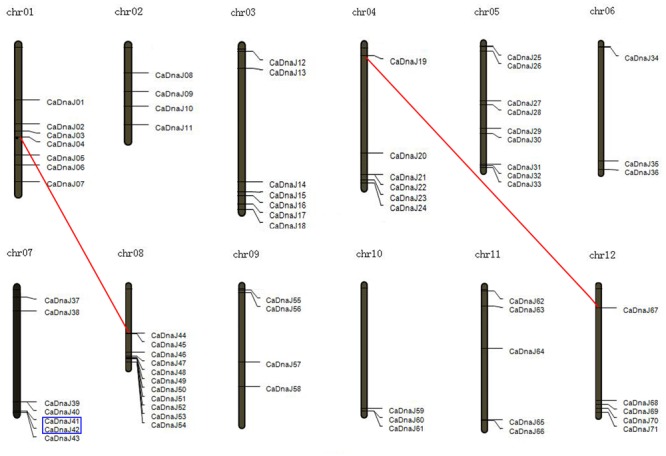
**Chromosomal map and duplication event coordinates of paralogous *CaDnaJ* gene candidates.** Segmental duplication and tandem duplication were indicated by red lines and blue boxes, respectively.

All these *CaDnaJ* genes (9 members in group A, 8 members in group B, 53 members in group C, 1 members in group D and 5 members in group E) in pepper were uneven distributed on 12 chromosomes (**Figure 1**). Among them, eleven and nine genes were located on chromosome 8 and 5, respectively. Seven genes on each of chromosome 1, 3, 7, six genes on chromosome 4, five genes on each of chromosome 11 and 12, four genes on each of chromosome 2, 9, three genes on each of chromosome 6 and 10.

We further analyzed the gene duplication of *CaDnaJ* genes in pepper. As shown in **Figure 1**, one tandem duplication event (CaDnaJ41/CaDnaJ42) was identified on chromosome 7. The chromosome location of this pair of *CaDnaJ* genes was close in distance and was inserted by less than one gene. In addition, two segmental duplication events were detected. CaDnaJ04 on chromosome 1 presented synteny to CaDnaJ44 localized on a duplicated segment of chromosome 8. Similar scenario was observed for CaDnaJ19 on chromosome 4 and CaDnaJ67 on chromosome 12. These results suggested that these duplication events made contributions to expansion of pepper *CaDnaJ* gene family.

### Analysis of Stress-Related *cis*-Elements in Pepper *CaDnaJ* Promoters

**FIGURE 2:**
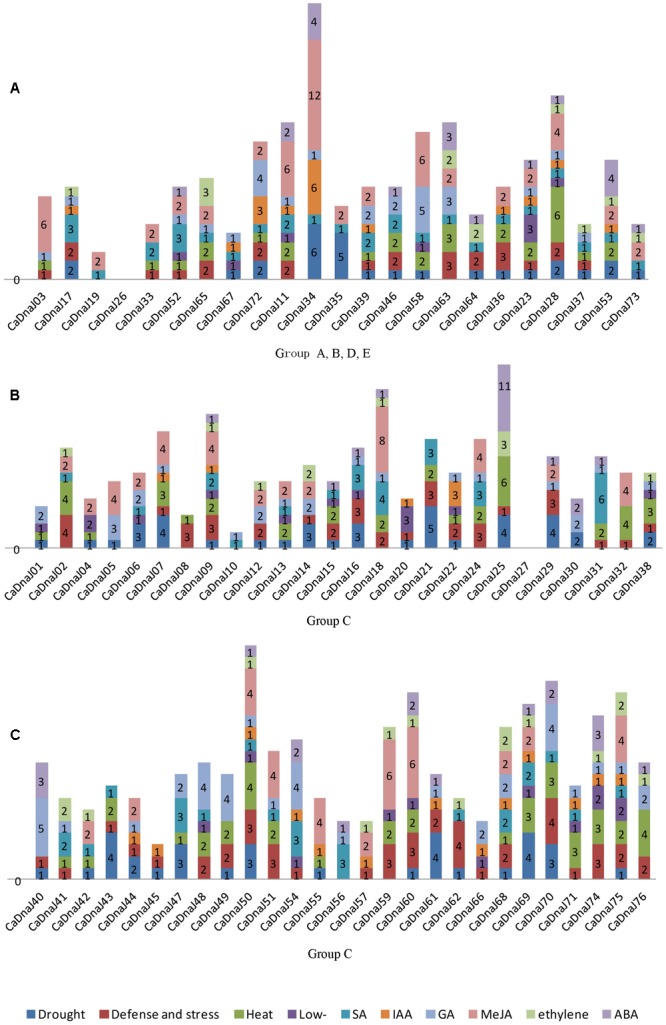
**Predicted *cis*-elements in the promoter regions of *CaDnaJ* genes.** Promoter sequences (–1500 bp) for 74 *CaDnaJ* genes (promoter regions of CaDnaJ26 and 27 were absent) are analyzed. The names of the promoters of *CaDnaJ* genes are shown on the bottom of the figure. Different *cis*-elements with the common functions are marked with same color. **(A)** Predicted *cis*-elements in the promoter regions of group A, B, D, E. **(B)** Predicted *cis*-elements in the promoter regions of group C (26 of 53 *CaDnaJs*). **(C)** Predicted *cis*-elements in the promoter regions of group C (27 of 53 *CaDnaJs*).

The upstream regions (1.5 kb) of the CaDnaJ sequences were searched for regulating factor on different stress conditions. The stress-related *cis*-elements were not been found in the promoter region of two *CaDnaJ* genes (CaDnaJ26 and 27), and the rest of 74 *CaDnaJ* genes possessed multiple *cis*-elements. Among them, the CaDnaJ 28 and 50 possessed the maximum types of stress-related *cis*-elements (10). On the contrary, only two types of stress-related *cis*-elements were held on CaDnaJ10. Besides, CaDnaJ 34 has the most stress-related *cis*-elements (23), including twelve methyl jasmonic acid (MeJA) related, six drought and indoleacetic acid (IAA) related, four ABA related and one GA related (**Figure 2** and **Supplementary Table S3**).

To further explore the possible regulation mechanism of *CaDnaJ* genes to HS, the heat stress responsiveness elements (HSEs) were also searched in the promoter region of all these *CaDnaJ* genes. The result showed that 66.21% (49 out of 74) *CaDnaJ* genes have HSEs in the promoter regions. The maximum numbers of HSEs (6) were identified in CaDnaJ25 and CaDnaJ28. Only one HSEs was found in fifteen *CaDnaJ* genes (CaDnaJ01, 03, 04, 08, 22, 33, 37, 39, 41, 42, 47, 52, 55, 68, and 72). In addition, other stress-related *cis*-elements were also detected. There were 107 TC-rich repeats in 55 genes, 99 MBS in 49 genes, 33 LTR in 26 genes, 79 TCA-element in 47 genes, 36 TGA-element in 27 genes, 87 GARE-motif in 49 genes, 138 CGTCA-motif in 41 genes, 37 ERE in 27 genes and 55 ABRE in 28 genes.

### Expression Patterns of *CaDnaJ* Genes in Different Tissues

**FIGURE 3:**
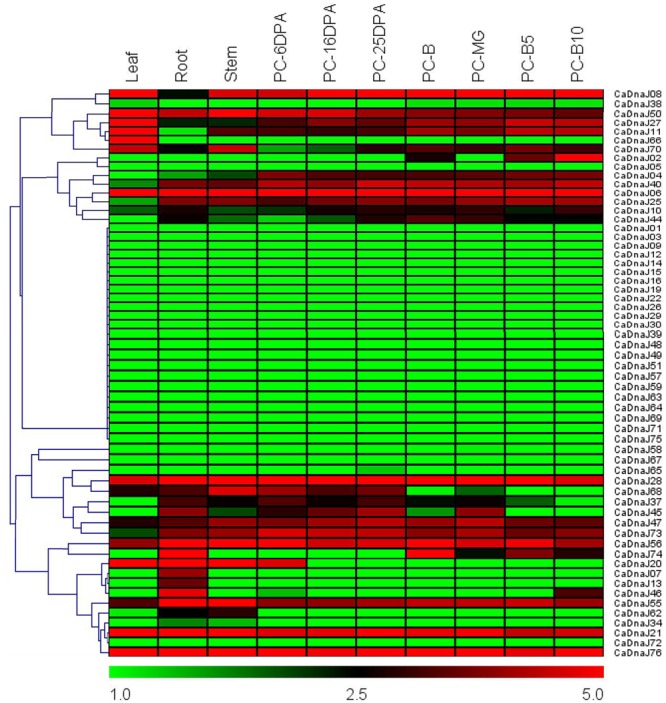
**Heat map of the expression profiles of *CaDnaJ* genes in various tissues.** The tested tissues are root, leaf, stem, and pericarp. PC, pericarp; MG, mature green; B, breaker; B5, 5 days post-breaker; B10, 10 days post-breaker; 6 DPA, 6 days post-anthesis; 16 DPA, 16 days post-anthesis; 25 DPA, 25 days post-anthesis. Fragments per kilobase of exon model per million mapped (FPKM) values were log2-transformed and heat maps with hierarchical clustering were exhibited using the software Mev 4.9.0.

Based on RNA-seq data of different pepper tissue (root, stem, leaf, and pericarp) published previously (Kim et al., 2014), expression profiles of *CaDnaJ* genes were revealed (**Figure 3**). A total of seven different stages of pericarp [6 DPA, 16 DPA, 25 DPA, MG, breaker (B), 5 days post-breaker (B5), B10] were selected for expression analysis in the present study. As shown in **Figure 3**, 29 out of 76 *CaDnaJ* genes were barely expressed in the tested tissues, including CaDnaJ01, 03, 05, 09, 12,14, 15, 16, 19, 22, 26, 29, 30, 38, 39, 48, 49, 51, 57, 58, 59, 63, 64, 65, 67, 69, 71, 72 and 75. The left 47 *CaDnaJ* genes could be detected at least in one tissue. Ten genes (CaDnaJ06, 08, 21, 27, 28, 47, 50, 55, 56 and 76) were expressed in all tested tissues. Of them, four genes (CaDnaJ06, 21, 28 and 76) were expressed at relatively high levels. Besides, tissue-specific expression was also found in some *CaDnaJ* genes. The CaDnaJ66 was specifically expressed in leaf, CaDnaJ07 and 13 were specifically expressed in root. During the stage of pericarp development, the expression of CaDnaJ02 was significantly up-regulated, while CaDnaJ74 and CaDnaJ45was obviously down-regulated.

### Expression Patterns of *CaDnaJ* Genes in Response to Heat Treatments

**FIGURE 4:**
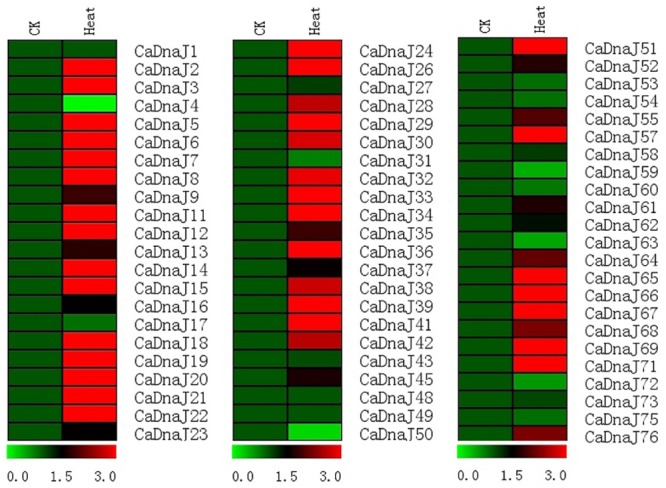
**Gene expression profiles of *CaDnaJ* genes in response to heat stress treatment.** The expression levels of these *CaDnaJs* under high temperature stress treatment were tested using qRT-PCR. Green, black, and red elements indicate low, regular, and high signal intensity, respectively.

To gain more insight into the role of *CaDnaJ* genes under HS condition, expression profiles of *CaDnaJ* genes in pepper response to high temperature based on qRT-PCR technique were performed. In this study, a total of 67 *CaDnaJ* genes were used to design successfully specific primers for expression analysis. As shown in **Figure 4**, expression of these tested *CaDnaJ* genes was significantly changed under high temperature stress treatments. Expression levels of five *CaDnaJ* genes (*CaDnaJ4, 50, 59, 63*, and *72*) were down-regulated, and 49 *CaDnaJ* genes were up-regulated. For the remaining 13 *CaDnaJ* genes (*CaDnaJ1, 17, 27, 31, 43, 48, 49, 53, 54, 58, 60, 73* and *75*), no difference was observed. Notably, among the *CaDnaJ* genes up-regulated, the expression levels of 71.4% (35/49) were increased to three folds. In total, expression of most of *CaDnaJ* genes was significantly altered under HS, indicating that the *CaDnaJ* genes were involved in plants response to high temperature stress.

## Discussion

As an important vegetable crop all over the world, pepper is deeply loved by a large population since its major ingredient in cuisines, essential vitamins and other healthy nutrients (Kim et al., 2014). High temperature has become one of the important environmental stresses and affected seriously the growth and development in pepper. The DnaJs, one of the significant Hsps, was produced in the process of plant responding to HS. Up to now, functional identification of DnaJ has been reported in many plant species (Miernyk, 2001; Qiu et al., 2006; Bekh-Ochir et al., 2013; Fristedt et al., 2014; Kong et al., 2014a,b). At the whole genome level, it has been found that *Arabidopsis* has 89 members and encoded multiple gene family (Miernyk, 2001). Recently, the whole pepper genome has released and provided an opportunity for identifying putative DnaJ homologous. In the current paper, a systematic analysis of CaDnaJ gene family was performed using bioinformatics methods, which focused on gene structure, chromosomal localization, stress-related *cis*-elements, and expression profiles in different tissues. The results would be significant for further analyzing the function of CaDnaJ members and illuminating the evolutionary relationship in pepper.

Since the first DnaJ proteins were isolated from *E. Coli* as 41 kDa Hsps (Georgopoulos et al., 1980), many DnaJ proteins have been subsequently reported in other life species (Bukau and Horwich, 1998; Miernyk, 2001; Walsh et al., 2004; Craig et al., 2006). Generally, DnaJ proteins contained one to four domains (J-domain, G/F-domain, zinc finger domains and less conserved C-terminal) (Silver and Way, 1993). Initially, the DnaJ proteins were classified into three groups (A, B, C) based on domain composition (Cheetham and Caplan, 1998; Miernyk, 2001). In this study, more complex structure of DnaJ genes was observed in pepper. For example, the HPD tripeptide which was crucial for J-domain function (Kampinga and Craig, 2010) was not found in CaDnaJ23, 28, 37, 53 and 73. Therefore, given the high diversity of *CaDnaJ* genes, a more systematic classification was proposed in our study. A total of 76 *CaDnaJ* genes were classified into five groups (A, B, C, D and E). Group A was characterized by the J-domain, zinc finger domains and a less conserved C-terminal. Group B were lack of the zinc finger domains but contained the J-domain and a less conserved C-terminal. Group C only comprised the J-domain. Group D would both have the J-domain and zinc finger domains but lack of C-terminal, and group E which have been described as J-like proteins with the J-domain lack of HPD motif (**Table 1** and **Supplementary Table S2**).

It was reported that gene duplication was become one of the primary evolution forces during the processes of genetic systems and genomes (Moore and Purugganan, 2003). In our study, chromosome location showed that all these *CaDnaJ* genes were mapped unevenly on 12 pepper chromosomes (**Figure 1**). Six of them were involved in gene duplication, including tandem duplication and segmental duplication (**Figure 1**). One tandem duplication event (*CaDnaJ41*/*CaDnaJ42*) and two segmental duplication events (*CaDnaJ04*/*CaDnaJ44*, and *CaDnaJ19*/*CaDnaJ67*) were observed. These results indicated that both tandem duplication and segmental duplication played role in expansion of the *CaDnaJ* gene family in pepper.

It has been demonstrated that *cis*-elements participated in responding multiple abiotic and biotic stresses. For instance, several *cis*-elements, such as ABRE, DRE, CRT, SARE and SURE, had been identified for responding to ABA, dehydration, cold, SA, and sulfur, respectively (Sakuma et al., 2002; Maruyama-Nakashita et al., 2005; Shi et al., 2010; Osakabe et al., 2014; Feng et al., 2016). In this paper, we identified *cis*-elements in the promoter regions of *CaDnaJ* genes using the PlantCARE server (Lescot et al., 2002). Two major groups of *cis*-elements were observed, including stress-responsive and hormone-responsive. The former contained HSE, LTR, and TC-rich *cis*-elements, which was responsive to heat, low-temperature, and defense, respectively. The latter was composed of TCA-element, TGA-element, GARE-motif, CGTCA-motif, ABRE and ERE, which was responsive to SA, IAA, GA, MeJA, ABA and ethylene, respectively (**Figure 2** and **Supplementary Table S3**). These results implied that *CaDnaJ* genes could be involved in the process of plant respond to multiple stresses. Especially, one of the most important *cis*-element, HSEs, which kept AAAAAATTTC as the core sequence, accounted for 13.64% of all the *cis*-elements. As we all known that the expression of Hsp was controlled by heat shock transcription factors that bind to HSEs in the promoter region of the Hsp genes (Hancock et al., 2009). A total of 49 (66.21%) *CaDnaJ* genes have HSEs in the promoter region. Thus, the results will contribute to further understand the vital function role of *CaDnaJ* genes under HS condition in the further.

To obtain more insights into the expression profiles of *CaDnaJ* genes in different tissues, RNA-seq data were acquired from leaf, root, stem and different stage of pericarp (Kim et al., 2014). Based on RNA-seq, we found that all these *CaDnaJ* genes exhibited three different expression patterns: (1) barely expression or too low expression level to detect; (2) constitutive expression; (3) tissue-specific expression patterns. The expression of these *CaDnaJ* genes differed in tissues tested, indicating that the CaDnaJ proteins may play different functional roles. Expression of some *CaDnaJ* genes showed tissue- and development-specific in root, stem, leaf and pericarp, suggested that they may participated in growth and development of pepper. In addition, we also found that 10 genes (CaDnaJ06, 08, 21, 27, 28, 47, 50, 55, 56 and 76) were highly expressed in all the tested tissues, implying that they might be involved in specific housekeeping action under normal growth conditions in pepper (**Figure 3**).

It has been known that plant growth and development were frequently affected by biotic and abiotic stresses under natural conditions (Xia et al., 2014). Previous researchers had reported that the DnaJ protein was involved in plant response to heat and drought stresses (Xia et al., 2014; Wang et al., 2015). To further comprehend the putative roles of *CaDnaJ* genes in pepper response to HS, expressions patterns of *CaDnaJ* genes under HS treatment conditions were analyzed by qRT-PCR. Expression analysis revealed that most *CaDnaJ* genes were changed in response to HS (**Figure 4**). Among them, almost half of these *CaDnaJ* genes were up-regulated for three folds. We also found that there are multiple types and numbers of *cis*-elements in these CaDnaJs promoter, which is reported to involved in abiotic stresses (Feng et al., 2016), including TC-rich, HSEs, MBS motif. All these results suggested that these *CaDnaJ* genes could involved in plant response to HS.

## Author Contributions

Conceived and designed the experiments: FF and YK. Performed the experiments: XY, XC, and YC. Analyzed the data: FF and YK. Wrote the paper: FF. All authors have read and approved the manuscript.

## Conflict of Interest Statement

The authors declare that the research was conducted in the absence of any commercial or financial relationships that could be construed as a potential conflict of interest.

## Footnotes

1 http://peppergenome.snu.ac.kr/

2 http://pfam.xfam.org/family/PF00226.29

3 http://pfam.xfam.org/

4 http://www.expasy.org/tools/protparam.html

5 http://chibba.agtec.uga.edu/duplication/

## Supplementary Material

TABLE S1 **Specific primer for qRT-PCR of each *CaDnaJ* gene**.

TABLE S2 **Multiple sequence alignment of the pepper CaDnaJs.** The sequences of 76 CaDnaJs were aligned according to the classification. Names of all the 76 members are showed on the left side of the figure. Conserved amino acid residues are highlighted in black. The sequences which boxed in red rectangle represented the conserved HPD motif, and sequences in yellow rectangle were zinc finger domains.

TABLE S3 **Stress-related *cis*-elements in pepper CaDnaJ promoters**.
