# Supplementary material for: The DnaJ Gene Family in Pepper (Capsicum annuum L.): Comprehensive Identification, Characterization and Expression Profiles
Source: Front Plant Sci. 2017 May 1;8:689. doi: 10.3389/fpls.2017.00689 (PMC5410566; doi:10.3389/fpls.2017.00689)
Supplement: TABLE S1 — Specific primer for qRT-PCR of each CaDnaJ gene. [file Table_1.DOC]

Supplementary Table S1 Specific primer for qRT-PCR of each CaDnaJ gene.

| Gene | Forward primer | Reverse primer |
| --- | --- | --- |
| CaDnaJ01  CaDnaJ02 | TGAGGAGGGAAGAGCTCCTGA  GCTGGTCATGCATCTGCTGT | TCGTCGAACGTCTCCAAGAGAA  CACTTGGCCGTTTGGACGAA |
| CaDnaJ03 | GATTTGCCTTGCACTGCTTGC | CCAGAACCTTTGCAGCGCTTA |
| CaDnaJ04 | GGCTTGTGTGCCAAAGGCTA | GGCCGCAGCTACCAGACTTA |
| CaDnaJ05 | AGGCTGCTAATGGCACAGTGA | GGCATTCCTGCGGGATCAAG |
| CaDnaJ06 | GAAAGCATTAGAGTTGCATCCAGACA | AGCTTCCTCGCTTTCTCATCCT |
| CaDnaJ07 | GCAAGACGAAGCGGAGATGG | CCCGCCTTCTTTCTGCATCC |
| CaDnaJ08 | AGCACCGGCGGAATTCTATGA | GCCGCTGTTGCTGTTGTGAA |
| CaDnaJ09 | TCGTGCGTCAGATTCCGTCA | AAACTGACAAACAGCTAGCAACACA |
| CaDnaJ11 | TGGAGAAGGCAGACCAATGAGT | AGTTCTCCCACTTGCATCAGCA |
| CaDnaJ12 | CGTAAATATCACCCAGAT | TCGAACCCTAATAAACCT |
| CaDnaJ13 | CGTCGATTCTATGATTGGACACTTGC | ACCAAGTCGGTCCACCATGT |
| CaDnaJ14 | AGGATTCCAGCAGAAAGCTCCA | AGGGCACCCGAGAAGCATTT |
| CaDnaJ15 | TGAAGCGTGGAGTTTGTTGTATGAC | ATTGGGCTGCGTAGAGGCAT |
| CaDnaJ16 | CTGGTCTCTCCGCTGCTGAA | CCTGAGTGTCACCCATGCCT |
| CaDnaJ17 | CGCCCTTCTTCTCATTCTCC | TAATCAGCGGCAGAGGAAAC |
| CaDnaJ18 | AGGAGTGCACTGAAACAGAGCTT | CTCCTCCACAAACTTCGAATTCCC |
| CaDnaJ19 | GATCCCGAGACGCGTGAGAT | TCTGCTGCTTCCACCACCTC |
| CaDnaJ20 | TTGAAGACTACATCGGACAT | AATTCAGAGGAAGCCATT |
| CaDnaJ21 | CTGGGTCCGGTTCCAGTTCA | CCTGAACCCTCACTGGTGCT |
| CaDnaJ22 | CAGCACAAGAGCCAGCGAAG | GCATTCGCGCAGTGTCAAGT |
| CaDnaJ23 | TTCAAGAATGCTACGGAGTT | CACCGACCAGGCTCTAAC |
| CaDnaJ24 | AACTCTCCTCCGCCTCTCCA | TAGCTCTGTCTGCGGTGGTG |
| CaDnaJ26 | GAGTGCGAGGAGACCACCTT | CACTCTCTGGGCACCACCAT |
| CaDnaJ27 | GTCACAATCCGGCCTCTA | TGCCTCCGCTAGTTCATT |
| CaDnaJ28 | CAATTCGCGTGATGCGGAAGA | TCCTCCAGACTACACGGCAAC |
| CaDnaJ29 | GCGAAACAATGAACTTGCCAGAGG | TCCCGCAGACCTTTCAGCTT |
| CaDnaJ30 | AGGCATACAGGAAAGCTGCCA | TGCGTGAGCCAACTCCTTGA |
| CaDnaJ31 | AAGAAAGTACCACCCAGAT | GTACCCGAATAAATCTCC |
| CaDnaJ32 | TGGACAGTAACAAAGACGAGGCT | CTTATTAAGGCGGCGCGCAA |
| CaDnaJ33 | GGTGGTGGACACGACCCATT | TTCCTCTGCTGCTTCCACCA |
| CaDnaJ34 | GTTCCAGCAGATGACAGA | GTAATGTGATAGCCCTCC |
| CaDnaJ35 | GAGGAGGTGGTGCGTTCAGA | AGTTCGAAGATACCCGGAAGCA |
| CaDnaJ36 | GTTTGGAAGAGCACCGAAGA | TTTGATGGCGGCTTTACG |
| CaDnaJ37 | ATCCTGTTGCTGCTGCGTCT | TGTGGTTGCTGCTGCTGTTG |
| CaDnaJ38 | GTCTCCAATTTGTCCTTTCAGTGGAG | CGTGCTTCCCTCTGCCTCTT |
| CaDnaJ39 | TCCAATCAACAATGTGGTTTCACCAA | CGCAGTAAGACGAGCAGGGA |
| CaDnaJ41 | TCATCCTGATTCTGCTGCTGCT | CTCGCCGTCGGGAACCTAC |
| CaDnaJ42 | GCAGCGTACCGGAATTTAGCG | TCCCTCGAAATAGGATCCGACAAC |
| CaDnaJ43 | ACGAGGCTGTCGATCGTTCTT | AGCACGTAGACCAGACACGAC |
| CaDnaJ45 | CAGATGCCAGCCATGCACTC | ACCGTCAGGAGCAGGAATGG |
| CaDnaJ48 | GCAGAACAACTTTACCCTCA | GGCTTCATCAGACGACCT |
| CaDnaJ49 | CCGTGCAGTAGTACCGTGGA | CGCTACCTGAGGGCGGTTTA |
| CaDnaJ50 | CTGCGCGAGCAACTTCTCAA | GTGCGTTCATGATGCTGCCA |
| CaDnaJ51 | CATCCGGATAGGTGGGCGAA | AAGGAAGCCGGCATCGTACA |
| CaDnaJ52 | TGGGCTTAAAGGAGCTGGCA | ACCTTCTGTGGCCCTGCTC |
| CaDnaJ53 | ACAGTCGCATCCGGTGAGTT | GCTGCGGAGAAGTCATTGGC |
| CaDnaJ54 | AAGATAACCAACGCAGAG | CAGGTCCATTTGTTCCAG |
| CaDnaJ55 | TGACGCATAAGACGGCATCCT | CACCGGAGGGCGTGGAATAA |
| CaDnaJ57 | TGCAGCAAGGCAGCAGTCTA | ACCCTCTGCATCTTCAGCAGT |
| CaDnaJ58 | TCTCCGAAGCCTACGACGTT | CACCACCACCACCACCATCT |
| CaDnaJ59 | TTTCACCCAGACAAACAC | CAATTAGAATCTCATACGC |
| CaDnaJ60 | AGAAATGGGCAAAGGAGG | CAACGGGCTTGTAGGTGG |
| CaDnaJ61 | TTAGGAGGCAGAGGTGGC | CCTTGTGGGAATGGAGCA |
| CaDnaJ62 | TGAGGAAGAGAAATGAGCTTCGACA | GCTCGTCGACAGACTGCTTC |
| CaDnaJ63 | AGGCGTATGATGTGCTTA | CAGTGCTAGTCGGAGATG |
| CaDnaJ64 | ATTTCGTTTGAAGGTAAGG | TGCTTGGACTAGAGGGAC |
| CaDnaJ65 | TGGAGAAGATGCACTGAAAGAAGGA | CACTACCACCACCTGCTCCA |
| CaDnaJ66 | GTTGAATCCGTTTGACTA | ACGATATTGCCTCTTGAC |
| CaDnaJ67 | GACGAGTGCGAGGAGACTACTT | CTCTCTGTGCACCACCACCA |
| CaDnaJ68 | CTCCTCTGTACCCACGAC | CACAATACTCACTTGCTCC |
| CaDnaJ69 | TGGAGATACAGAGAGCCTGGGA | TCGGCAGTTGCTGTATCCTGT |
| CaDnaJ71 | CCAGACAGGTTTGGTTGAGGAATTT | GCTGTCCTAAACATATCAGAACGACCA |
| CaDnaJ72 | AGGTTCGAAGAGTGGAGCATCTG | CTGAGCCTCGGCATTGAGGA |
| CaDnaJ73 | TCAGGGTCAAATGCTGCTGGA | ATCGAAGTCACAGGCTGCGT |
| CaDnaJ75 | TGTCACTACAGCTAGGCAAGATGC | AGCAGCTTCAGCACGATGTC |
| CaDnaJ76 | CTGGTCTCTCCGCTGCTGAA | CCTGAGTGTCACCCATGCCT |
