# Supplementary material for: The DnaJ Gene Family in Pepper (Capsicum annuum L.): Comprehensive Identification, Characterization and Expression Profiles
Source: Front Plant Sci. 2017 May 1;8:689. doi: 10.3389/fpls.2017.00689 (PMC5410566; doi:10.3389/fpls.2017.00689)
Supplement: TABLE S2 — Multiple sequence alignment of the pepper CaDnaJs. The sequences of 76 CaDnaJs were aligned according to the classification. Names of all the 76 members are showed on the left side of the figure. Conserved amino acid residues are highlighted in black. The sequences which boxed in red rectangle represented the conserved HPD motif, and sequences in yellow rectangle were zinc finger domains. [file Table_2.DOC]

CaDnaJ03 **----------** **----------** **----------** **----------** **----------** **----------** **----------** **----------** **MSSKDFYEVL** **GVNRNATASE** **IKKAYYGLAK**

CaDnaJ17 **---MACTISL** **SLRPSSHSPS** **LPPS------** **-SSSSSFFSS** **-----GWAHT** **KFMSLSVSFS** **SSSSQYNKKI** **AVRRFGRLVV** **SSAADYYSTL** **GVPKSANSKE** **IKSAYRRLAR**

CaDnaJ19 **----------** **----------** **----------** **----------** **----------** **----------** **----------** **-MFGRAPKK-** **SENTKYYEIL** **GVPKTAAQKD** **LKKVYRKAAI**

CaDnaJ26 **----------** **----------** **----------** **----------** **----------** **----------** **----------** **-MFGRAPKK-** **SDNNKYYEIL** **GVAKTATQDD** **LKKAYRKLAI**

CaDnaJ33 **----------** **----------** **----------** **----------** **----------** **----------** **----------** **-MFGRAPKK-** **SDNTKYYEIL** **GVPKTAAPED** **LKKAYRKAAI**

CaDnaJ52 **MAIIPCGSTW** **VARWGVQPQF** **MPKSFTVTNK** **LSASPFCFSS** **KIRALASPNS** **TFFCQESLQA** **LFSSVSNKNH** **YQRRGARLVV** **RAEKDYYDIL** **GVSRNASKSE** **IKSSYRKLAR**

CaDnaJ65 **----------** **----------** **----------** **----------** **----------** **----------** **----------** **-MFGRGGKKQ** **SDNTRYYEIL** **GVSKNAKEDE** **IKKAYRKAAM**

CaDnaJ67 **----------** **----------** **----------** **----------** **----------** **----------** **----------** **-MFGRAPKK-** **SDNTKYYDIL** **GVQKSASQDD** **LKKAYRKAAI**

CaDnaJ72 **----------** **----------** **----------** **----------** **----------** **----------** **----------** **-MFGWTPKR-** **SDNSKYYEVL** **GVSKSASQDE** **LKKAYRKAAI**

CaDnaJ03 **RLHPDMNKDD** **PEAEKKFQEV** **SKAYEVLKDD** **KTREQYDQLG** **HDAFNNMNNG** **GGGGPGFDPF** **GGFKSPFEDI** **FRNADIFGNI** **F------NRD** **MGGEDVKVPI** **ELSFMEAVQG**

CaDnaJ17 **QYHPDVNKE-** **PDAPEKFKEI** **KDAYEVLSDD** **KKRALYDQYG** **EAGVKSSVGA** **QTGAYTTNPF** **DLFETFFGSS** **MGGFGMDGAG** **FGTRR-RSTV** **TKGEDLRYDV** **TLEFSASIFG**

CaDnaJ19 **KNHPDKGGD-** **---PEKCKEL** **AQAYEVLSDP** **ETREIYDQYG** **EDALKQGMG-** **-GGGGGHDPF** **DIFSSFFG--** **GSPFGGGGGS** **SRGRR----Q** **RRGEDVVHPL** **KVSLEDLYNG**

CaDnaJ26 **KNHPDKGGD-** **---PEKFKEI** **AQAYDVLSDP** **EKREIYDQYG** **EDALKEGMD-** **-RSGG-HDPF** **DIFQSFFG--** **GSAFGGGG-S** **SRGRR----Q** **RRGEDVVHSL** **KVSLEDLYNG**

CaDnaJ33 **KNHPDKGGD-** **---PEKFKEL** **AQAYEVLSDP** **EKREIYDQYG** **EDALKEGMG-** **-GGGGGHDPF** **DIFSSFFG--** **GSPFGGGGGS** **SRGRR----Q** **RRGEDVVHPL** **KVSLEDLYNG**

CaDnaJ52 **SYHPDVNKE-** **PGAEQKFKEI** **SNAYEVLSDD** **EKRSVYDKYG** **EAGLKG-AGM** **GMGDFS-NAF** **DLFESLFDG-** **FGGMGGMGGG** **MGGRSSRSRA** **TEGEDQGYNL** **VLNFKEAVFG**

CaDnaJ65 **KNHPDKGGD-** **---PEKFKEL** **AQAYEVLSDS** **QKREIYDQYG** **EDALKEGMG-** **-GGGGMHDPF** **DIFESFFG--** **GNPFGAGGGS** **GRGRR----Q** **RRGEDVVHPL** **KVSLEDLYSG**

CaDnaJ67 **KNHPDKGGD-** **---PEKFKEI** **AQAYDVLSDP** **EKREIYDQYG** **EDALKEGMG-** **-GGGGGHDPF** **DIFQSFFGGG** **GSPFGGGG-S** **SRGRR----Q** **RRGEDVIHPL** **KVSLEDLYNG**

CaDnaJ72 **RNHPDKGGD-** **---PEKFKEL** **VQAYEVLSDP** **NKRHIYDQYG** **EDALKEGMGT** **-GSGGVHNHF** **DIFHSFFG--** **-GSFGGGGSH** **FRASR----Q** **KRGEDVVHTL** **GVTLEDLYNG**

CaDnaJ03 **CSKTITFQTD** **LPCTACGGTG** **VPPGTKPETC** **KRCKGSG--V** **SISQT--GPF** **TLQTTCPSCK** **GTGKIVSSF-** **--CKSCKGNR** **VLRGPKTVKV** **DIMPGVDTDE** **TLKVYGSGGA**

CaDnaJ17 **AEKEFDLSHL** **ETCEVCMGTG** **AKVGSKMRIC** **STCGGRGQVM** **RTEQTPFGMF** **SQVSVCPKCG** **GDGEMISEY-** **--CRKCSGEG** **RVRVKKNIKV** **KIPPGVSKGS** **ILRVAGEGDA**

CaDnaJ19 **TSKKLSLSRN** **VLCPKCKGKG** **SKS-------** **---GSSMKFT** **IRQLGPSMIQ** **QMQHPCNECK** **GTG-EMINDK** **DRCGQCKGEK** **VVQEK-VLEV** **VVEKGMQNGQ** **KITFPGEADE**

CaDnaJ26 **TSKKLSLSRN** **VLCTKCKGVG** **SKSGASMKCS** **GCQGKGMKVT** **IRQLGP-MIQ** **QMQHPCNECK** **GTG-EKINDK** **DRCPQCKGQK** **VVQEKKVLEV** **VVDKGMQNGQ** **KITFPGEADE**

CaDnaJ33 **TSKKLSLSRN** **VLCPKCKGKG** **SKSGASMKCS** **GCQGSGMKVT** **IRQLGPSMIQ** **QMQHPCNECK** **GTG-EMINDK** **DRCGQCKGEK** **VVQEKKVLEV** **VVEKGMQNGQ** **KITFPGEADE**

CaDnaJ52 **VEKEIEISRL** **ETCGTCDGSG** **AKPGTKPSTC** **NTCGGQGQVV** **SSARTPLGVF** **QQVTTCSSCG** **GTG-EISTP-** **--CNTCSGDG** **RVRKSKRISL** **KVPPGVDSGS** **RLRVRSEGNA**

CaDnaJ65 **ITKKLSLSRN** **VICSKCSGKG** **SKSGASTKCS** **GCKGTGMKVS** **IRQLGPGMIQ** **QMQHPCNECK** **GTG-ETIDDK** **DRCPQCKGEK** **VVPEKKVLEV** **HVEKGMQNGQ** **KITFPGEADE**

CaDnaJ67 **TSKKLSLSRN** **VLCSKCKGKG** **SKSGASMKCS** **GCQGSGMKVS** **IRQLGPSMIQ** **QMQHPCNECK** **GTG-ETISDK** **DRCPQCKGEK** **VVQEKKVLEV** **HVEKGMQNGQ** **KVTFPGEADE**

CaDnaJ72 **ATKKLSLSRN** **ILCPKCKGKG** **SKSGASGACY** **GCQGTGVRVT** **TRHIAPGMIQ** **QMQHVCPQCR** **GSGREFISER** **DRCLQCNGYK** **VTLEKKVLEV** **NVEKGMENNQ** **EITFKGEADE**

CaDnaJ03 **DPEGNRPGDL** **YVVFKVREDP** **VFRREGSDIH** **VDAVLSITQA** **ILGGTIQVPT** **LT--------** **--------GD** **VVVKVLKYY-** **----------** **----------** **----------**

CaDnaJ17 **GPRGAPPGDL** **YVYLEIEEIP** **EIQRDGINLI** **STVSVGYLDA** **ILGTVVKVKT** **VEG---VTDL** **QIPPGTQPGD** **VLVLARKGAP** **KLNRPSIRGD** **HLFTIKVSIP** **KR--IRCASV**

CaDnaJ19 **AP-DTVTGDI** **VFVLQQKEHP** **KFKRKGDDLF** **VEHTLSLTKA** **LCGFQFFLTH** **LDS-------** **----------** **----------** **----------** **----------** **----------**

CaDnaJ26 **AP-DTITGDI** **VCILQQKEHP** **KFKRKGDDLF** **VEHTLTLTEA** **LCGFQFVLTH** **LDNRQLMIKS** **QPGEVIKPDQ** **FKGINDEGMP** **LYQSSFMRGK** **LYIHFTVDFP** **DA--LTPELC**

CaDnaJ33 **AP-DTVTGDI** **VFVLQQKEHP** **KFKRKGDDLF** **VEHTLSLTEA** **LCGFQFILTH** **LDNRQLIIKS** **QPGEVVKPDQ** **FKAINDEGMP** **MYGRPFMRGK** **LYIHFTVEFP** **DT--LSPDQC**

CaDnaJ52 **GRRGGPPGDL** **FVMIEVLPDP** **VLKRDDTNIL** **FNCKVSYIDA** **ILGTTMKVPT** **VDG---MVDL** **KIPAGTQPGT** **TLVMAKKGVP** **LLSKPNMRGD** **QLVRVQVEIP** **KR--LSSEER**

CaDnaJ65 **AP-DTVTGDI** **VFVLQQKEHP** **RFKRKGEDLF** **VDHTLSLTEA** **LCGFHFILTH** **LDGRQLLIKS** **NPGEVVKPDQ** **FKAINDEGMT** **VYQRPFMKGK** **LYIHFIVEFP** **DS--LSPEQV**

CaDnaJ67 **AP-ETITGDI** **VFVLQQKEHP** **KFKRKGDDLF** **VEHTLTLTEA** **LCGFQFVLTH** **LDNRQLLIKS** **QPGEVVKPDQ** **FKAINDEGMP** **MYQRPFMRGK** **LYIHFTVDFP** **ES--LTPEQC**

CaDnaJ72 **AP-DTITGDI** **FIVLQQKEHP** **KFKTDFSDLH** **VEHTLTLTEA** **LCGFQFVLTH** **LDGRQLLIKS** **NPGEVIKPDQ** **CKAINGEGMP** **KFRMHFIKGR** **LYIHFKVVFP** **ESGVLSPEKC**

CaDnaJ03 **----------** **----------** **----------** **----------** **----------** **----------** **----------**

CaDnaJ17 **KLYMMN----** **----------** **----------** **----------** **----------** **----------** **----------**

CaDnaJ19 **----------** **----------** **----------** **----------** **----------** **----------** **----------**

CaDnaJ26 **KNLEAVLPQK** **PKTQATDMEL** **DECEETTLHD** **VNIEEEMRRK** **QQQQAQEAYE** **EDDDDMHGGA** **QRVQCAQQ--**

CaDnaJ33 **KNLEAVLPAK** **PKTQMTDMEL** **DECEETTLHD** **VNIEEEMRRK** **QQ-QAQEAYD** **EDDEDMHGGA** **QRVQCAQQ--**

CaDnaJ52 **KLIEELANLN** **KPKAATNSKR** **----------** **----------** **----------** **----------** **----------**

CaDnaJ65 **QALEAILPAR** **PKSQYSDMEL** **DECEETTLHD** **VNMEEEMRRK** **QA-AQQEAYD** **EDEEMPGGGA** **QRVQCAQQ--**

CaDnaJ67 **KNLEAVLPPK** **PKQQVSDMEL** **DECEETTLHD** **VNIEDEMRRK** **QQ-AAQEAYD** **EDDDMHGGGA** **QRVQCAQQ--**

CaDnaJ72 **RSIEAILPTS** **PGKSSLEMEL** **DDCEETIMHD** **VNIEEEMRRK** **AQRRRQEAYD** **SDNDEPH--M** **HGVACNQQ--**

Group A

CaDnaJ11 **----------** **----------** **---MGVDYYK** **ILGVDKNAKD** **DDLKKAYRKL** **AMKWHPDKNP** **-NNKKEAEAK** **FKQISEAYEV** **VLSDSQKRAI** **YDQYGEEGLK** **-GQVPPPDAT**

CaDnaJ34 **MAEGRSKLLF** **LLCILSSSLI** **ISIAAKSYYD** **ILQVPKGASD** **EQIKRAYRKL** **ALKYHPDKNQ** **--GNEEANKK** **FAEISNAYE-** **VLSDGDKRNI** **YDRYGEEGLK** **QHAASGGRGS**

CaDnaJ35 **----------** **----------** **---MGVDYYN** **ILKVNRNASE** **EDLRKAYRRL** **AMIWHPDKNL** **GNNKYEAEAK** **FKQISEAYD-** **VLSDPQKRQI** **YDLYGEEALK** **TGQVPTPPRG**

CaDnaJ39 **----------** **----------** **---MGVDYYK** **VLGVDRNASD** **DDLKKAYRKL** **AMKWHPDKNP** **-NNKKDAEAK** **FKQISEAYD-** **VLSDSQKKTV** **YDQFGEEGLK** **-GGVPPPGAG**

CaDnaJ46 **----------** **----------** **---MGVDYYN** **VLNVVKTATE** **DDLKKAYRKL** **AMKWHPDKNP** **-NNKKEAEAQ** **FKQISEAYE-** **ILSDPDKRQI** **YDQYGEEGLK** **--EMPSPGCS**

CaDnaJ58 **----------** **----------** **---MGVDYYK** **VLQVDRNAKD** **DDLKKAYRKL** **AMKWHPDKNP** **-NNKKEAESK** **FKQISEAYD-** **VLSDPQKRVV** **YDQYGEEGLK** **-GQVPPPGAG**

CaDnaJ63 **----------** **----------** **---MGIDYYN** **ILKVSRNASE** **EDLKRSYKRL** **AMKWHPDKNN** **-QNKKEAEAK** **FKQISEAYD-** **VLSDVQKRQI** **YDLYGEDALK** **SGQFASPTST**

CaDnaJ64 **----------** **----MGESTK** **SSMPASKYYG** **ILEISKSASL** **ADICKSYKHL** **VRKWHPDRNR** **-SNQAEAVEK** **FRSINEAYR-** **VLSQNKSEEV** **DILKNDAAKT** **PKNFLKQQEK**

CaDnaJ11 **GGPGGATFFH** **TGEGPNVFRF** **NPRNANDIFA** **EFFGFSSPFG** **GGMGGGPGGG** **MGGGPGGMRG** **TRFS-SSMFG** **DDIFG-SFG-** **----EGRPMS** **-----SGSRK** **APPIERTLPC**

CaDnaJ34 **G---------** **----------** **--MNMQDIFS** **QFFGGGTMEE** **EEEKIVK---** **----------** **---------G** **DDVIVDLDAT** **LEDLYMGGSL** **KVWREKNILK** **PAPGKRRCNC**

CaDnaJ35 **GPHNMRNPHP** **----NPSFRF** **NPRDADDIYA** **ELFGSSSSAA** **GESSSG----** **-RGRKG----** **---------R** **DGFFRSTANG** **GAEFSGVGNS** **GTGGGGAFRK** **AAPVENVLLC**

CaDnaJ39 **GPGAGSTYFS** **AGEGATPFRF** **NTRNADDIFA** **EFFGFSPPFG** **APGG------** **--GRTS----** **-RFG--SAFT** **DDIFASSFGE** **GGGGGGASMH** **Q----SAPRK** **EAPIQQNLPC**

CaDnaJ46 **G---------** **----------** **HPRNAEDIFA** **EFFGSSPFGF** **GSTGVK----** **-STRFS----** **---------S** **EGSAFAGFGG** **GENIFRTASN** **GTG-ANMPKK** **PPPVESKLPC**

CaDnaJ58 **GFPG-SSDGG** **GGGGHGSFRF** **NPRSADDIFS** **EFFGFSAPFG** **G-MG-----D** **MGGRTG---A** **SGFSRGSMFG** **EDIFT-SFRN** **A---AGEGAS** **G----NASRK** **AAPLERALPC**

CaDnaJ63 **GSGSGN----** **----GRGFRF** **NTRDAEDIFA** **EFFGGSD---** **GYSSS-----** **----------** **----------** **----------** **----SGVGID** **--------KK** **AAPVENKLPC**

CaDnaJ64 **DEDDNELQIS** **SPILLSRTTS** **RISPSIDFYT** **SMPSFLSISG** **ASTPTTP---** **----------** **----------** **-RTPISDQTP** **SLSKITSKPI** **IFSQSTSRRK** **PQPIKKKLEC**

CaDnaJ11 **SLEELYK--G** **TTKKMKISRE** **IADASG--RT** **LPVEEILTID** **IKPGWKKGTK** **ITFPEKGNEE** **PNVAPADLMF** **IIDEKPHSVF** **TRDGNDLVTN** **QRISLAEALA** **GYTVHLTTLD**

CaDnaJ34 **RNEVYHRQIG** **PGMFQQMTEQ** **VCDQCPNVKF** **EREGYHITVD** **IEKGMQDGQE** **VVFYEDGEPK** **IDGEPGDLKF** **RIRTAPHDRF** **KREGNDLHTT** **VTITLAQALV** **GFNKTVKHLD**

CaDnaJ35 **SLEDLYK--G** **AKKKMKISRT** **VLDASG--YL** **RTLEEILTID** **IKPGWKKGTK** **VTFPEKGNEE** **PGIIPADLVF** **VIEEKPHHVY** **ARDGNDLTVN** **QEISLLEALT** **GKTLELMTLD**

CaDnaJ39 **SLEDIYK--G** **ATKKMKISRE** **VADASG--KR** **MQVQEILTIN** **IKPGWKKGTK** **ITFQGKGNEY** **PGVIPADLVF** **ILDEKPHKVF** **SRDGNDLIVT** **HKISLIEALT** **GSTVHLTTLD**

CaDnaJ46 **SLEELYS--G** **STRKMKISRT** **VVDKNG--WL** **VTESEILTID** **VKPGWKKGTK** **ITFPDKGNEQ** **LNQLPADLVF** **VIDEKPHDVY** **KRDGNDLIRN** **YKVTLAEALG** **GKTVNLTTLD**

CaDnaJ58 **SLEDLYK--G** **TTKKMKISRE** **VTDAAG--RP** **STTEEILTID** **IKPGWKKGTK** **ITFPEKGNEQ** **RGIIPSDLVF** **IIDEKPHGVF** **KRDGNDLIIT** **QKISLVEALT** **GYTAQITTLD**

CaDnaJ63 **SLEELYK--G** **SKRKMKISRI** **LLDDSG--KP** **TTVEEVLAIH** **IKPGWKKGTK** **ITFPEKGNYE** **HGAAPGDLVF** **VIEEKPHAVF** **KRDGNDLVIN** **QKISLLDALT** **GKTIGLTTLD**

CaDnaJ64 **TLEELCN--G** **CVKKVTITRD** **FIATTG--LI** **IKEEEVLTIK** **VKPGWKRGTK** **ISFEGKGEER** **AGTHGSDIIF** **SIDEKKHSLF** **KREGDDLVLG** **VEVPLVQALT** **GCTITIPLLG**

CaDnaJ11 **GRKLTIPIN-** **NVIHPNYEEV** **VPREGMPIPK** **DPSKRGNLRI** **KFNIKFPARL** **TAEQKTGIKK** **LLTS------**

CaDnaJ34 **DHLVDISTN-** **GITEPKKVRK** **FKGEGMPLHF** **N-NKKGDLYV** **KFEVLFPTSL** **TEDQKKKVKE** **ILV-------**

CaDnaJ35 **GRNLNIPLT-** **DIVKPGHEIV** **VRNEGMPISR** **EPRKKGNLRI** **KMDVNYPTRL** **TEAQKSDLRR** **VLGGSS----**

CaDnaJ39 **GRNLTIPIN-** **NVVSPTSEHV** **VQGEGMPLPK** **DPSKKGNLRI** **KFDIKFPARL** **TATQKSGLKE** **LLSGS-----**

CaDnaJ46 **SRELTIPVN-** **QIVRPGYELV** **VAKEGMPITK** **EPGNRGDLKI** **KFDVKFPTRL** **SAEQKAALKR** **ALG-------**

CaDnaJ58 **GRVLTIPIN-** **SVISPNYEEV** **IKGEGMPIPK** **EPSRKGNLRV** **KFNIKFPSKL** **TSEQKAGIKR** **YLT-------**

CaDnaJ63 **ERKLTIPIT-** **EVVKPGQELI** **IPNEGMPISK** **ERGNKGNLKI** **KFDIRFPSRL** **SADQKSDLRR** **VLCRSID---**

CaDnaJ64 **GDEMTVSFDD** **KIIYPGYEKI** **IPGQGMPKPK** **QENIRGDLVL** **QFLIEFPIYL** **SEDQRFQVVS** **ILEDHITSE-**

Group B

CaDnaJ01 **DHYSALGLMR** **F----QSIDA** **S-VLKREYRK** **KAMLVHPDKN** **MG--------** **----------** **-------NAK** **AAEA------** **--------FK** **KLQNAYEVLL** **DSLKRKAYD-**

CaDnaJ02 **NFYGILGVS-** **-----PKADD** **E-AIRKQYRK** **LALMLHPDKN** **------KSI-** **----------** **---------G** **AEAA------** **--------FK** **HVSEAWSLLS** **DKTKKTAYD-**

CaDnaJ04 **DWYGILQID-** **-----MVADQ** **V-TVKKQHRR** **LALVLHPDKN** **------KFP-** **----------** **---------G** **AEAA------** **--------FK** **LIGEANMVLS** **DPTKRAMYD-**

CaDnaJ05 **DWYSILKVD-** **-----PTADD** **A-LIRKQFRK** **FALSLHPDKN** **------KFP-** **----------** **---------G** **AADA------** **--------FT** **LIGDAQAVLL** **DRQQRMLYN-**

CaDnaJ06 **DHYAVLDLPS** **GEEGAKLSEK** **D--ISKAYKK** **KALELHPDKR** **RD--------** **----------** **---------D** **PNAHL-----** **------NFQN** **-LKTSYEILK** **DEKARKLFD-**

CaDnaJ07 **DYYKILQVE-** **-----RFSRT** **D-TIKTQYKK** **LALALHPDKN** **------PFV-** **----------** **---------A** **SEEA------** **--------FK** **VVGEAFRVLS** **DKIRRKEYD-**

CaDnaJ08 **DLYDLLGVE-** **------SSSN** **QAQIKLAYRM** **LQKRCHPDIA** **G---------** **----------** **----------** **PSGHDM----** **--------AI** **ILNEVYALLS** **DPIARMAYD-**

CaDnaJ09 **-FYEVLGIP-** **------IGAK** **IGEIKTAYRR** **LARVCHPDES** **YAD-------** **----------** **--------EF** **MKVHAA----** **--------FM** **KVHAAYCTLS** **DPGKRADYD-**

CaDnaJ10 **NAYEVLGVS-** **------ETSS** **FAEIKDSFRK** **LAKETHPDLA** **HST-------** **----------** **--------HT** **SSSSNR----** **--------FL** **EILAAYEILS** **DSVKRAHYD-**

CaDnaJ12 **-FYELLGIP-** **------ETVS** **LFEIKEAYKQ** **LVRKYHPDVS** **PPD-------** **----------** **--------RV** **EEYTQR----** **--------FI** **RVREAYETLS** **DPMSRDMYD-**

CaDnaJ13 **-HYQFLGVS-** **------ATAD** **LEEIKAAYRR** **LSKEYHPDTT** **----------** **----------** **----------** **NLPIRT----** **----ASEKFM** **KLREIYDVLS** **DEEQRRFYD-**

CaDnaJ14 **DWYGVLGVS-** **-----PSTDD** **E-TVRKQYRK** **LALILHPDKN** **------KSV-** **----------** **---------G** **AEGA------** **--------FK** **LLSEAWSLLS** **DKSKRLAYN-**

CaDnaJ15 **DIYRILSVE-** **-----PSVDD** **E-TIRKQYRR** **LSLALHPDKN** **------KSV-** **----------** **---------G** **ADGA------** **--------FK** **IISEAWSLLY** **DSRR------**

CaDnaJ16 **DPFSILGLEP** **G-----VSDS** **A--IKKAYRR** **LSIQYHPDKN** **P---------** **----------** **---------D** **PAAHK-----** **------YFVE** **YISKAYQALT** **DPISRENF--**

CaDnaJ18 **DFYAVLGLK-** **-----KECTE** **T-ELRNAYKK** **LAMKWHPDRC** **SASGNSKFVE** **----------** **---------E** **SKKK------** **--------FQ** **VIQEAYSVLS** **DANKRFLYD-**

CaDnaJ20 **EYYDVLGVSP** **-----LASEE** **E--IRKAYYL** **KARQVHPDKN** **PD--------** **----------** **---------D** **PLAAQ-----** **------RFQ-** **----------** **----------**

CaDnaJ21 **DWYSILQIQG** **-----RTEDS** **E-LIKKQYRR** **LALLLHPDKN** **------KYP-** **----------** **---------S** **SDVA------** **--------FG** **LVADAWAVLS** **DPNKKGLYD-**

CaDnaJ22 **DHYVVLNLPS** **GEKGSMLSQH** **D--ISKAYKK** **KALELHPDKR** **PN--------** **----------** **---------D** **PNAHL-----** **------DFQK** **-LKTSYDILK** **DEKLRKSYD-**

CaDnaJ24 **-LYEVLGIQ-** **------TGAN** **FHEIKSAYRK** **LARVLHPDVV** **KLQ-------** **----------** **--------Q-** **NSSAEE----** **--------FI** **RVQSAYATLS** **DPEKRANYD-**

CaDnaJ25 **DFYLILAIK-** **-----SSDTE** **S-DIKKAYRK** **AALRHHPDKA** **GQ----SFAR** **SDTVDDGGLW** **KEISETVRND** **ADRL------** **--------FK** **LIGEAYAVLS** **DTDKRAKHD-**

CaDnaJ27 **DPYEVLSVR-** **------KDSS** **DQEIKSAYRK** **LALKYHPDKN** **S---------** **----------** **----------** **NNPE------** **----ASELFK** **EVAYSYGILS** **DPEKRRQYD-**

CaDnaJ29 **DFYTILGMD-** **-----PSANR** **A-KLKKQYKR** **MAMLLHPDKN** **------KTV-** **----------** **---------G** **ADGA------** **--------FR** **FVSEAWTVLS** **DRAKRSSYD-**

CaDnaJ30 **-LYEVLGVP-** **------KNSS** **PDDIKKAYRK** **AAIKNHPDKG** **----------** **----------** **----------** **GDPE------** **----K---FK** **ELAHAYEVLS** **DPEKREIYN-**

CaDnaJ31 **TFYDLLGIP-** **------VTGS** **LLEIKQAYKQ** **LARKYHPDVS** **PPD-------** **----------** **--------RV** **EEYTRR----** **--------FI** **RVQEAYETLS** **DPGMRALYD-**

CaDnaJ32 **DYYEILGLEK** **C-----CSVD** **E--IRKAYRR** **ISLKVHPDKN** **M---------** **----------** **---------A** **PGYAD-----** **------EF--** **----------** **----------**

CaDnaJ38 **DFYAILGLD-** **-----TSVDK** **A-KLKKQYKK** **MAVLLHPDKN** **------KSV-** **----------** **---------G** **ADGA------** **--------IR** **LVSEAWTVLS** **DGAKRSSYD-**

CaDnaJ40 **DFYAVLGLL-** **------PDAT** **PAQIKKAYYN** **CMKSCHPDLS** **GD--------** **----------** **---------D** **PETTNF----** **--------CM** **FINEVYEILS** **DPVQRRVYD-**

CaDnaJ41 **-LYDVLRVK-** **------RDAS** **AKEIKAAYRH** **LAKLYHPDSA** **AAT-------** **----------** **--------LP** **EEVSDGR---** **-------NFI** **EIHDAYVTLS** **DPSARALYD-**

CaDnaJ42 **-FYDVLRVK-** **------ENAS** **VKEIKAAYRN** **LAKVYHPDVA** **CR--------** **----------** **---------P** **EEYSDDR---** **-------NFI** **EIHEAYATLS** **DPISRDLYD-**

CaDnaJ43 **DHYEALGISR** **H----KKIDT** **L-LLKKEYRK** **KAMLVHPDKN** **MG--------** **----------** **-------SAL** **ASES------** **--------FK** **KLQCAYEVLS** **DSVKKRDYD-**

CaDnaJ44 **DWYGILQID-** **-----MVADE** **V-TIKKQYRR** **LALVLHPDKN** **------KFP-** **----------** **---------G** **AEAA------** **--------FK** **LIGEANMVLS** **DPTKRALYD-**

CaDnaJ45 **-------EN-** **------IDE-** **-EKLKRQYRK** **LAMKYHPD--** **----------** **----------** **----------** **KNPE------** **----GREKFL** **AVQKAYERL-** **----------**

CaDnaJ47 **DHYALLGLSH** **L----RYLAS** **EDQIRKSYRD** **AALRHHPDKL** **AS--------** **----------** **-------LLL** **AEETEASKQA** **KKEEIENHFK** **AIQEAYEVLI** **DPIRRRIYD-**

CaDnaJ48 **DWYGILQLD-** **-----RSSDE** **A-NIKKQYRR** **LALMLHPDKN** **------EFP-** **----------** **---------G** **AEAA------** **--------FK** **LIAEAHMVLS** **DQVKRSLYD-**

CaDnaJ49 **DWYGILQLD-** **-----RSSDE** **A-TIKKQYRR** **LALMLHPDKN** **------KLP-** **----------** **---------G** **AEAA------** **--------FK** **LIVEAHMVLS** **DQVKRSLYD-**

CaDnaJ50 **-AYDVLGVA-** **------PNCS** **ADELKSAFRN** **KVKKCHPDVR** **R---------** **----------** **--------DG** **NSSDKM----** **--------IR** **RVIQAYEMLS** **NLTK------**

CaDnaJ51 **-YYSVLGIR-** **-----KDASC** **S-DIRSAYRK** **LALKWHPDRW** **AK--NPTVAG** **----------** **---------E** **AKRR------** **--------FQ** **KIQEAYSVLS** **DQDKRSMYD-**

CaDnaJ54 **-PYEVLGVS-** **------SSAS** **ADEIKRAYRK** **LALKYHPDVN** **----------** **----------** **----------** **KEHN------** **----AQEKFM** **RIKHAYSTLL** **NSKTRKRYD-**

CaDnaJ55 **-PFSILGVEY** **G-----ASDS** **E--IKKAYRR** **LSIQYHPDKN** **P---------** **----------** **---------D** **PEAHS-----** **------YFVE** **FISKAYQALT** **DPVSRENF--**

CaDnaJ56 **DWYKILGVS-** **-----KTASV** **S-EIKKAYKK** **LALQWHPDKN** **VDNR------** **----------** **--------EE** **AENK------** **--------FR** **EIAAAYEVLG** **DEDKRTRYD-**

CaDnaJ57 **----------** **----------** **----------** **LSLQYHPDKN** **KN--------** **----------** **--------KG** **AQEK------** **--------FA** **EINNAYEILS** **DEDKRRNYD-**

CaDnaJ59 **DHYKVLGLT-** **-----KSASK** **E-EIKQAFRK** **LAMEFHPDKH** **AHS-------** **----------** **------SNQL** **KENAT-----** **------FKFK** **QVSEAYEILI** **DDRKRADYN-**

CaDnaJ60 **-LYEILGVET** **-----TASQQ** **E--IKKAYYK** **LALRLHPDKN** **PD--------** **----------** **---------D** **EEAKE-----** **------KFQQ** **-LQKVISILG** **DEEKRALYD-**

CaDnaJ61 **-HYSVLGVP-** **------HNAS** **SIDIKKAYRL** **LALKYHPDVS** **----------** **----------** **----------** **KDPG------** **----ADEVFK** **KIHLAYDVLS** **DESSRNQYD-**

CaDnaJ62 **DPYEVLGVS-** **------KDSS** **DQEIKTAYRK** **LALKYHPDKN** **A---------** **----------** **----------** **NNPE------** **----ASELFK** **EVAYSYSILS** **DPEKRRQYD-**

CaDnaJ66 **NPFDYLNLS-** **------FDSS** **IDEVKRQYRK** **LSLLVHPDKC** **----------** **----------** **----------** **KHPQ------** **----AKEAFG** **ALAKAQQLLL** **DPQER-----**

CaDnaJ68 **DYYDILGLEK** **G-----CSVE** **E--VRKAYRK** **LSLKVHPDKN** **K---------** **----------** **---------A** **PGAEE-----** **------AFKM** **-VSKAFKCLS** **DEESRRTYD-**

CaDnaJ69 **THYEILGVK-** **------EDAN** **FEEVRKAYRS** **AILCFHPDKQ** **QNA-------** **----------** **--------SE** **TSNSECVT--** **-----DNKFL** **EIQRAWETVG** **NPRSRALYD-**

CaDnaJ70 **EYYDILGVSP** **-----TATEA** **E--IKKAYYI** **KARQVHPDKN** **PN--------** **----------** **---------D** **PLAAQ-----** **------NFQV** **-LGEAYQVLS** **DPSQRQAYD-**

CaDnaJ71 **DYYAILGLEK** **G-----CTVE** **E--IRKSYRK** **LSLKVHPDKN** **K---------** **----------** **---------A** **PGSEE-----** **------AFKK** **-VSKAFKCLS** **DDDSRRQYD-**

CaDnaJ74 **-YYSVLGVN-** **-----VDSSD** **E-EIRRAYRK** **LAMQWHPDKW** **TR--TPSLLG** **----------** **---------E** **AKRK------** **--------FQ** **QIQEAYSVLS** **DQNKRMMYD-**

CaDnaJ75 **DIYGVLSVE-** **-----PSADN** **E-TIRKNYRR** **LALALHPDKN** **------KSV-** **----------** **---------G** **ADGA------** **--------FK** **IISEAWSLLS** **DRKKRMMYD-**

CaDnaJ76 **DPFSILGLEP** **G-----VSDS** **A--IKKAYRR** **LSIQYHPDKN** **P---------** **----------** **---------D** **PAAHK-----** **------YFVE** **YISKAYQALT** **DPISRENF--**

Group C

CaDnaJ36

MFGRAPKKSD NTKYYDILGV QNRASQDDLK KAYRKAAIKN HPDKGGDPEK FKEIAEAYDV

LRDPEKREIY AQYGEDALKE GMGAGGGRHD PFDIFQSFFG GGGSSRGRRQ RRGEDVTPPL

KVSLEDLYNG ASKKLSLSRN VFLKCSGCQG SGMKVSIRLL GPSMIQQRQH PCNECKGTGE

TISDKNRCPQ CNGEGSCRRR RCWKFMWRRV SRTGRR

Group D

CaDnaJ23 **----------** **----------** **----------** **----------** **----------** **----------** **------MRSN** **TVN-EHPNYY** **SVLQVP-NYT** **HDHQVILNSF** **KNATELLNPN**

CaDnaJ28 **----------** **----------** **----------** **----------** **----------** **----------** **----------** **----MGVGYY** **KILKVSRHAS** **E--EDLKKSY** **K---------**

CaDnaJ37 **MEHPFFINGP** **-PTNRSEALR** **WLSIAEKLLT** **NRDLVGSKSF** **ATRARESDPS** **LAHPIDQILS** **IVDTLTAGDK** **RINNHHFDYY** **SILQVPPNQT** **QNFEFIADQY** **RRFALLLNPQ**

CaDnaJ53 **MEHPFFMNGA** **GNTSRAEAIR** **WLSIAEKLLT** **NRDLVGSKSF** **AIRAHESDPT** **LLP-ADQILA** **IVDTLIAGDK** **RINNQHLDYY** **SILQIPSNQT** **HDSELIANQY** **RRLALLLNPQ**

CaDnaJ73 **MEHPFFLNAA** **-PANRAEALR** **LLQIAEKLLN** **NRDLVGSKSF** **ATRARESDPT** **LSPVTDQILA** **ILDTLIAGDK** **RINNHHFDYY** **SILRIPSSQT** **QNVDFIAEQY** **RRFAVLLDPQ**

CaDnaJ23 **VNRYPLASEV** **FRVVVRAWSV** **LSNQIQKKQF** **DDELRKME--** **----------** **----------** **----------** **----------** **----------** **----------** **----------**

CaDnaJ28 **---QKEAEVK** **FKQISEAYDV** **LSDSQKRQIY** **DIYGDEALK-** **----------** **----------** **----------** **----------** **----------** **----------** **----------**

CaDnaJ37 **NNTFPFSDQA** **FGLVVDAFSV** **LSDPMRKSMY** **DKELGFFINL** **YPVAAASAAS** **VPTSVSFVQQ** **QQQQPQHSGS** **AYGHMQGSNT** **ADQLFVNMPS** **QDSQG-----** **SFSRDPQ-TG**

CaDnaJ53 **KNNFPFADHA** **FQLVVDAWAV** **LSNAFRRSVY** **DKEIGFFLNL** **NPVSSPPTPP** **PPPAAPNNAS** **IGFMQHS---** **---MIFQSQQ** **QSHPVSSVPS** **SSRERQ--TV** **TFLQDPQQQP**

CaDnaJ73 **SNSFPFTEQA** **CRLVFDAYSV** **LSNPMRKNMY** **DKELGFFRNL** **YPVVGQ----** **--------NQ** **NVCMP-----** **----MPINSN** **ADQVFVNLPS** **QDSGSNAAGL** **SFSRDPQ-AG**

CaDnaJ23 **----------** **----------** **----------** **----------** **----------** **-KDGRFFDPT** **RKSCLGDGGW** **SFLNPTQKTH** **FDNGLKS---** **----------** **----------**

CaDnaJ28 **----------** **----------** **----------** **----------** **----------** **SGQFDPSSPS** **MNGTYGNGRG** **FKFNSRDAED** **IFAEFFG---** **----------** **----------**

CaDnaJ37 **ISSMPMPVTF** **MG--------** **----SSGSEQ** **EQQQQQQQPP** **VMSMRQQQAQ** **QQQQPPVSSL** **SFSNIDEQPA** **TFLNLNQPQP** **-VSSERSLNR** **ENP-------** **----------**

CaDnaJ53 **MTSPQQPVGF** **LGRIQTQPVS** **STMLSPGREQ** **NPFTFGLSST** **RGQQQVAFAE** **STRGQQQAAF** **VESVRGPQQV** **AFAESTRAQH** **RVASMESRRE** **KQEVAPVHQQ** **GNKQAPQRTE**

CaDnaJ73 **IS---MPMSS** **MA--------** **----SSGMEQ** **Q--------P** **ATFLR-----** **-QQTQPVTSI** **SFSKADEQPA** **TFLNLNQPQP** **-VNSVRSLNR** **ENQQFGTGSS** **STQGREQVVS**

CaDnaJ23 **----------** **GSFSARPKPG** **WMMG------** **----------** **----------** **----------** **----------** **----------** **----------** **----------** **----------**

CaDnaJ28 **----------** **GSEGYSRSTG** **GSIRIRKAAP** **AEN-------** **----------** **---KLPCSLE** **ELYKRSKRK-** **----------** **----------** **--------MK** **ISRIVLDGTG**

CaDnaJ37 **----------** **PPFGIGLSST** **--RGRE----** **----------** **----------** **---PEVVSVE** **QQRGKQQPP-** **----------** **----------** **---------E** **RRNENVVGNN**

CaDnaJ53 **GFVGNNQNYS** **ASIGRNVNNE** **GLFGSQQNHS** **ASISKNVNNE** **GLFGNNQNQS** **ATISKNVNSE** **GLFGNNQNQS** **----ATVSKN** **VNNEGLFGNH** **QNQSASINRN** **VNNQGLFGNN**

CaDnaJ73 **VEKHGHQREN** **PQFGVGSSSK** **--QGREQVVS** **AEKHGNQRGN** **PQFGIGSSST** **QGGEQVVSAE** **QRGNQQEAPQ** **FGIGSSSPQG** **REEERVHVFF** **AEQHGNKLAQ** **KGNENMVGNN**

CaDnaJ23 **----------** **----------** **----------** **----------** **----------** **----------** **----------** **----------** **----------** **----------** **----------**

CaDnaJ28 **----------** **----------** **---KPTTVEE** **VLAIHIKHGW** **KKGTKITFPE** **KGNHEPGAAP** **GDLIFVIDEK** **LHDVFKRDGN** **DLVINQKISL** **VDALAGKTIN** **LTTFDGRELT**

CaDnaJ37 **E---------** **----------** **-NTRAASTSN** **NNVKEKEGRV** **DGSESRNIPS** **FWTACPYCYV** **MHEYPLEYVD** **CTLKCQNCRR** **AFQAVKVAAP** **P-IVDGKEAY** **FCCWGFMPLG**

CaDnaJ53 **QNHSANTSHS** **NVNNAGLFGN** **YQKHSVSTSI** **NNVNNVGKGA** **DAS-SHAVPS** **FWTACPYCYV** **MYEYPLVYVD** **CTLRCQKCKR** **AFQAVQIASP** **PPTIDGQDAY** **ICCWGHMPLG**

CaDnaJ73 **----------** **----------** **-ASKSASASE** **N-VKEKEGNA** **DAS-GKKIPS** **FWTACPCCLS** **MYEYSVDYTN** **RYLSCQNCNK** **AFQAVPIASP** **PPIVDGKELN** **FCSWGFMPFG**

CaDnaJ23 **----------** **----------** **----------** **----------** **----------** **----------** **----------** **----------** **----------** **----------** **----------**

CaDnaJ28 **IPITDVVKPG** **HEQIIPNEGM** **PI--------** **----------** **----------** **----------** **----------** **----------** **----------** **----------** **----------**

CaDnaJ37 **FSVESYQRSR** **NNVSSWSPFS** **PMFAVPSSGV** **NEGRKANNHA** **VGGQGNVSTY** **GNLHNAGGSR** **SRAGRKQSAP** **RMIYSDDDDD** **DDDVLVDIS-** **----------** **----------**

CaDnaJ53 **FNMDVFKKYK** **GNISSWTPFA** **PMFNKQ--GV** **SKSPAPRTYI** **D---DYEDVF** **LGLSESSEES** **DEDWKGDNKV** **KKAKSGKRKS** **K---------** **----------** **----------**

CaDnaJ73 **LCLEDFNRN-** **IDSSSWSPFS** **PMFTCPRFGG** **NGGGNVKNHA** **VGGQSNVNKL** **GSLHNAGGSV** **SGVGRKNSAP** **TIYIVDDEED** **D--VFVEVSG** **SDEEWSQVKE** **RKKAKNGKRK**

CaDnaJ23 **----------** **----------** **----------** **----------** **----------** **----------** **----------** **----------** **----------** **----------** **----------**

CaDnaJ28 **----------** **----------** **----------** **----------** **----------** **----------** **----------** **----------** **----------** **----------** **----------**

CaDnaJ37 **----EVLSDD** **A---------** **----------** **----------** **----------** **----------** **----------** **----------** **----------** **----------** **----------**

CaDnaJ53 **----RLRKKK** **AKIPKTDKGK** **KVVGNAGDNM** **QDVSVTQGGV** **EMPNVTTAQS** **SKRGIGGNTR** **R-AGRVAKDV** **GKLDLNVEFS** **NEVEEPLAPG** **MGQGNGAGTG** **EDGNIEGIEF**

CaDnaJ73 **SARTGTPSKN** **AKKQQAGKTE** **TVEGKNGVSL** **QDGLATQGGV** **EMSHVVAVES** **SKRGVASKTR** **RHPGRFAKHF** **GNLDLNVEFS** **NEVEEPKIQ-** **--MSNVAGEG** **DDDTVEVIGF**

CaDnaJ23 **----------** **----------** **----------**

CaDnaJ28 **----------** **----------** **----------**

CaDnaJ37 **----------** **----------** **----------**

CaDnaJ53 **FEGLDEFLSS** **LPILNAVGDD** **KVKAA-----**

CaDnaJ73 **FDGLDEFLSG** **LPILDAVDGD** **KIEAA-----**

Group E
