# Supplementary material for: The DnaJ Gene Family in Pepper (Capsicum annuum L.): Comprehensive Identification, Characterization and Expression Profiles
Source: Front Plant Sci. 2017 May 1;8:689. doi: 10.3389/fpls.2017.00689 (PMC5410566; doi:10.3389/fpls.2017.00689)
Supplement: TABLE S3 — Stress-related cis-elements in pepper CaDnaJ promoters. [file Table_3.doc]

**Supplementary Table S3 | Stress-related cis-elements in pepper CaDnaJ promoters**

| Group | Gene | Drought | Defense and stress | Heat | Low-temp | SA | IAA | GA | MeJA | Ethylene | ABA |
| --- | --- | --- | --- | --- | --- | --- | --- | --- | --- | --- | --- |
| A | CaDnaJ03 |  | 1 | 1 |  |  |  | 1 | 6 |  |  |
| A | CaDnaJ17 | 2 | 2 |  |  | 3 | 1 | 1 |  | 1 |  |
| A | CaDnaJ19 |  |  |  |  | 1 |  |  | 2 |  |  |
| A | CaDnaJ26 |  |  |  |  |  |  |  |  |  |  |
| A | CaDnaJ33 |  | 1 | 1 |  | 2 |  |  | 2 |  |  |
| A | CaDnaJ52 |  | 1 | 1 | 1 | 3 |  | 1 | 2 |  | 1 |
| A | CaDnaJ65 |  | 2 | 2 |  | 1 |  | 1 | 2 | 3 |  |
| A | CaDnaJ67 | 1 |  |  | 1 | 1 | 1 | 1 |  |  |  |
| A | CaDnaJ72 | 2 | 2 | 1 |  | 1 | 3 | 4 | 2 |  |  |
| B | CaDnaJ11 |  | 2 | 2 | 1 | 2 | 1 | 1 | 6 |  | 2 |
| B | CaDnaJ34 | 6 |  |  |  | 1 | 6 | 1 | 12 |  | 4 |
| B | CaDnaJ35 | 5 |  |  |  | 1 |  |  | 2 |  |  |
| B | CaDnaJ39 | 1 | 1 | 1 |  | 2 | 1 | 2 | 2 |  |  |
| B | CaDnaJ46 | 1 | 2 | 2 |  | 2 |  | 2 |  |  | 1 |
| B | CaDnaJ58 | 1 |  | 2 | 1 | 1 |  | 5 | 6 |  |  |
| B | CaDnaJ63 |  | 3 | 3 |  | 1 |  | 3 | 2 | 2 | 3 |
| B | CaDnaJ64 | 1 | 2 |  |  | 1 |  |  |  | 2 | 1 |
| C | CaDnaJ01 | 1 |  | 1 | 1 |  |  | 2 |  |  |  |
| C | CaDnaJ02 |  | 4 | 4 |  | 1 |  |  | 2 | 1 |  |
| C | CaDnaJ04 | 1 |  | 1 | 2 |  |  |  | 2 |  |  |
| C | CaDnaJ05 | 1 |  |  |  |  |  | 3 | 4 |  |  |
| C | CaDnaJ06 | 3 |  |  | 1 | 1 |  | 2 | 2 |  |  |
| C | CaDnaJ07 | 4 | 1 | 3 |  |  | 1 | 1 | 4 |  |  |
| C | CaDnaJ08 |  | 3 | 1 |  |  |  |  |  |  |  |
| C | CaDnaJ09 | 1 | 3 | 2 | 1 | 2 | 1 |  | 4 | 1 | 1 |
| C | CaDnaJ10 |  |  |  |  | 1 |  | 1 |  |  |  |
| C | CaDnaJ12 | 1 | 2 |  |  |  |  | 2 | 2 | 1 |  |
| C | CaDnaJ13 | 1 |  | 2 | 1 | 1 |  | 1 | 2 |  |  |
| C | CaDnaJ14 | 3 | 1 |  |  |  |  | 2 | 2 | 2 |  |
| C | CaDnaJ15 | 1 | 2 | 2 | 1 | 1 |  |  |  |  | 1 |
| C | CaDnaJ16 | 3 | 3 |  | 1 | 3 |  | 1 |  |  | 1 |
| C | CaDnaJ18 |  | 2 | 2 |  | 4 |  | 1 | 8 | 1 | 1 |
| C | CaDnaJ20 | 1 | 1 |  | 3 |  | 1 |  |  |  |  |
| C | CaDnaJ21 | 5 | 3 | 2 |  | 3 |  |  |  |  |  |
| C | CaDnaJ22 | 1 | 2 | 1 | 1 |  | 3 | 1 |  |  |  |
| C | CaDnaJ24 |  | 3 | 2 |  | 3 |  | 1 | 4 |  |  |
| C | CaDnaJ25 | 4 | 1 | 6 |  |  |  |  |  | 3 | 11 |
| C | CaDnaJ27 |  |  |  |  |  |  |  |  |  |  |
| C | CaDnaJ29 | 4 | 3 |  |  |  |  | 1 | 2 |  | 1 |
| C | CaDnaJ30 | 2 |  |  |  |  |  | 2 |  |  | 2 |
| C | CaDnaJ31 |  | 1 | 2 |  | 6 |  | 1 |  |  | 1 |
| C | CaDnaJ32 |  | 1 | 4 |  |  |  |  | 4 |  |  |
| C | CaDnaJ38 | 2 | 1 | 3 | 1 |  |  | 1 |  | 1 |  |
| C | CaDnaJ40 | 1 | 1 |  |  |  |  | 5 |  |  | 3 |
| C | CaDnaJ41 |  | 1 | 1 |  | 2 |  | 1 |  | 2 |  |
| C | CaDnaJ42 | 1 |  | 1 |  | 1 |  |  | 2 | 1 |  |
| C | CaDnaJ43 | 4 | 1 | 2 |  | 1 |  |  |  |  |  |
| C | CaDnaJ44 | 2 | 1 |  |  |  | 1 | 1 | 2 |  |  |
| C | CaDnaJ45 | 1 | 1 |  |  |  | 1 |  |  |  |  |
| C | CaDnaJ47 | 3 |  | 1 |  | 3 |  | 2 |  |  |  |
| C | CaDnaJ48 |  | 2 | 2 | 1 | 1 |  | 4 |  |  |  |
| C | CaDnaJ49 | 1 | 2 | 2 |  |  |  | 4 |  |  |  |
| C | CaDnaJ50 | 3 | 3 | 4 | 1 | 1 | 1 | 1 | 4 | 1 | 1 |
| C | CaDnaJ51 |  | 3 | 2 |  | 1 |  | 1 | 4 |  |  |
| C | CaDnaJ54 |  | 1 |  | 1 | 3 | 1 | 4 |  |  | 2 |
| C | CaDnaJ55 | 1 |  | 1 |  |  | 1 |  | 4 |  |  |
| C | CaDnaJ56 |  |  |  |  | 3 |  | 1 |  |  | 1 |
| C | CaDnaJ57 |  | 1 |  |  |  | 1 |  | 2 | 1 |  |
| C | CaDnaJ59 |  | 3 | 2 | 1 |  |  |  | 6 | 1 |  |
| C | CaDnaJ60 | 1 | 3 | 2 | 1 |  |  |  | 6 | 1 | 2 |
| C | CaDnaJ61 | 4 | 2 |  |  |  | 1 | 1 |  |  | 1 |
| C | CaDnaJ62 | 1 | 4 |  |  | 1 |  |  |  | 1 |  |
| C | CaDnaJ66 |  | 1 |  | 1 |  | 1 | 2 |  |  |  |
| C | CaDnaJ68 | 1 | 2 | 1 |  | 2 | 1 | 2 | 2 | 2 |  |
| C | CaDnaJ69 | 4 |  | 3 | 1 | 2 | 1 |  | 2 | 1 | 1 |
| C | CaDnaJ70 | 3 | 4 | 3 |  | 1 |  | 4 |  |  | 2 |
| C | CaDnaJ71 |  | 1 | 3 | 1 | 1 | 1 | 1 |  |  |  |
| C | CaDnaJ74 |  | 3 | 3 | 2 |  | 1 | 1 |  | 1 | 3 |
| C | CaDnaJ75 | 1 | 2 | 2 | 2 | 1 | 1 | 1 | 4 | 2 |  |
| C | CaDnaJ76 |  | 2 | 4 |  |  |  | 2 |  | 1 | 1 |
| D | CaDnaJ36 | 1 | 3 | 2 |  | 1 | 1 |  | 2 |  |  |
| E | CaDnaJ23 | 1 | 1 | 2 | 3 | 1 | 1 | 1 | 2 |  | 1 |
| E | CaDnaJ28 | 2 | 2 | 6 | 1 | 1 | 1 | 1 | 4 | 1 | 1 |
| E | CaDnaJ37 | 1 | 1 | 1 |  | 1 |  | 1 |  | 1 |  |
| E | CaDnaJ53 | 2 |  | 2 |  | 1 | 1 |  | 2 | 1 | 4 |
| E | CaDnaJ73 | 1 |  |  |  | 1 |  |  | 2 | 1 | 1 |

SA, salicylic acid; IAA, auxin; GA, gibberellin; MeJA, methyl jasmonate; ABA, abscisic acid.
